# Recruiting population controls for case-control studies in sub-Saharan Africa: The Ghana Breast Health Study

**DOI:** 10.1371/journal.pone.0215347

**Published:** 2019-04-16

**Authors:** Sarah J. Nyante, Richard Biritwum, Jonine Figueroa, Barry Graubard, Baffour Awuah, Beatrice Wiafe Addai, Joel Yarney, Joe Nat Clegg-Lamptey, Daniel Ansong, Kofi Nyarko, Seth Wiafe, Joseph Oppong, Isaac Boakye, Michelle Brotzman, Robertson Adjei, Lucy T. Afriyie, Montserrat Garcia-Closas, Louise A. Brinton

**Affiliations:** 1 University of North Carolina at Chapel Hill, Chapel Hill, NC, United States of America; 2 University of Ghana, Accra, Ghana; 3 University of Edinburgh, Edinburgh, Scotland; 4 National Cancer Institute, Rockville, MD, United States of America; 5 Komfo Anokye Teaching Hospital, Kumasi, Ghana; 6 Peace and Love Hospital, Kumasi, Ghana; 7 Korle Bu Teaching Hospital, Accra, Ghana; 8 Ghana Statistical Service, Accra, Ghana; University of Arkansas for Medical Sciences, UNITED STATES

## Abstract

**Background:**

In case-control studies, population controls can help ensure generalizability; however, the selection of population controls can be challenging in environments that lack population registries. We developed a population enumeration and sampling strategy to facilitate use of population controls in a breast cancer case-control study conducted in Ghana.

**Methods:**

Household enumeration was conducted in 110 census-defined geographic areas within Ghana’s Ashanti, Central, Eastern, and Greater Accra Regions. A pool of potential controls (women aged 18 to 74 years, never diagnosed with breast cancer) was selected from the enumeration using systematic random sampling and frequency-matched to the anticipated distributions of age and residence among cases. Multiple attempts were made to contact potential controls to assess eligibility and arrange for study participation. To increase participation, we implemented a refusal conversion protocol in which initial non-participants were re-approached after several months.

**Results:**

2,528 women were sampled from the enumeration listing, 2,261 (89%) were successfully contacted, and 2,106 were enrolled (overall recruitment of 83%). 170 women were enrolled through refusal conversion. Compared with women enrolled after being first approached, refusal conversion enrollees were younger and less likely to complete the study interview in the study hospital (13% vs. 23%). The most common reasons for non-participation were lack of interest and lack of time.

**Conclusions:**

Using household enumeration and repeated contacts, we were able to recruit population controls with a high participation rate. Our approach may provide a blue-print for others undertaking epidemiologic studies in populations that lack accessible population registries.

## Introduction

Case-control is one of the most efficient study designs for studying rare diseases, such as breast cancer. There are multiple types of control groups that can be employed in a case-control study, each with advantages and disadvantages [1]. Most prior investigations of breast cancer risk in sub-Saharan Africa have used hospital-based controls [2–8], a combination of hospital and hospital visitor controls [9, 10], or patients with cancers at sites other than the breast [11, 12]. Hospital-based or cancer patient controls can be appealing: there are readily available subject rosters, contact information, and health histories, as well as greater motivation to participate than the general public. At the same time, hospital-based controls may introduce bias if they are not representative of the population from which breast cancer patients are obtained. Furthermore, it can be difficult in a hospital setting to select controls with conditions that are unrelated to the study risk factors of interest. The relationship of hospital controls to the case population base can also be difficult to assess in sub-Saharan Africa, given the often not well-characterized hospital referral patterns, widespread use of traditional healers, self-treatment through pharmacies, and cultural stigmas which may prevent some from seeking care.

In a study with a well-defined study base, the use of population controls may avoid some of the potential for bias associated with the use of hospital-based controls. Controls sampled directly from a geographically-defined study area are not dependent on referral or treatment-seeking patterns and, therefore, may be more likely to represent the general population from which the cases arise, assuming complete ascertainment of cases [1]. One prior study of breast cancer risk in sub-Saharan Africa recruited population controls by sampling participants from the community adjacent to study hospital [13], but, to our knowledge, there have been no breast cancer studies where controls were sampled from multiple communities across a diverse geographic region. In the Ghana Breast Health Study (GBHS)[14], we identified population controls by enumerating defined geographic areas within and surrounding the two largest cities in Ghana, where case recruitment was based. Our objective in this manuscript was to describe, in detail, our approach to recruiting GBHS population controls. We report the GBHS population enumeration and control recruitment strategies, participant retention at each step, and enrolled participant characteristics. We also describe measures taken during the recruitment process to increase participation. Our experiences have the potential to inform strategies for similar recruitments conducted in other parts of the world.

## Materials and methods

The GBHS was approved by the Special Studies Institutional Review Board of the National Cancer Institute (Rockville, MD, USA), the Ghana Heath Service Ethical Review Committee and institutional review boards at the Noguchi Memorial Institute for Medical Research (Accra, Ghana), the Kwame Nkrumah University of Science and Technology (Kumasi, Ghana), the School of Medical Sciences at Komfo Anokye Teaching Hospital (Kumasi, Ghana), and Westat (Rockville, MD, USA). All participants provided written informed consent.

### Study design

**Fig 1:**
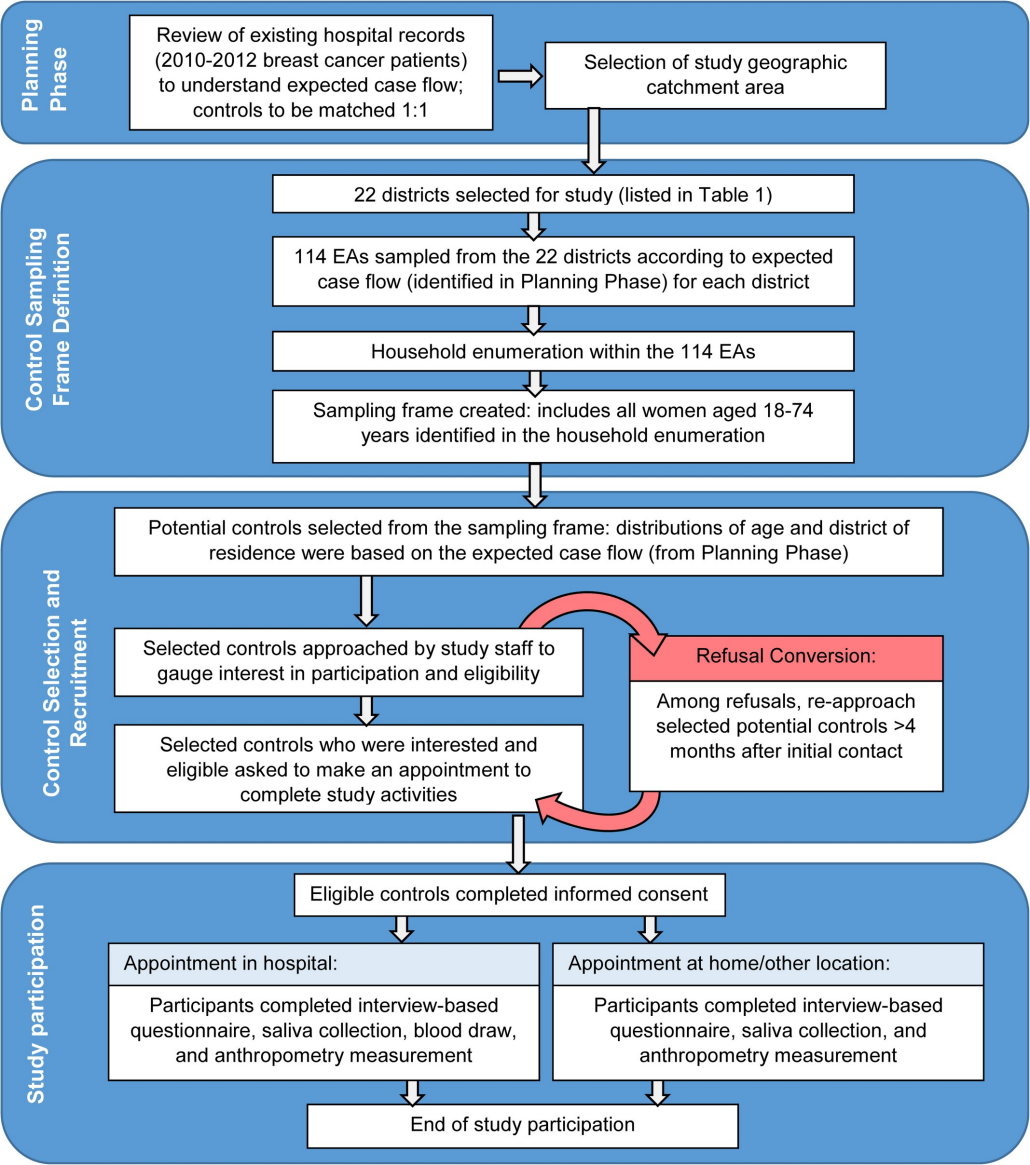
Overview of the control participant identification and recruitment process in the Ghana Breast Health Study. Four distinct phases were involved in the identification and recruitment of study controls. As shown in the figure, existing records were used to estimate the study catchment area and the number of controls that would be needed. Household enumeration was used to generate a pool of potential controls (*i*.*e*., the sampling frame) from which women were selected and approached for study participation.

An overview of the activities involved in the enumeration and recruitment of GBHS controls is shown in Fig 1. The GBHS utilized a case-control design that has been described previously [14]. Briefly, eligibility requirements for all study participants (cases and controls) included being: (i) female; (ii) 18 to 74 years old; (iii) a resident within one of the 22 defined municipal districts for at least one year prior to enrollment; and (iv) able to complete an in-person interview in either English or Twi language. Eligible cases were defined as: women being recommended for biopsy of a breast lesion suspicious for malignancy at one of the three major cancer treatment hospitals in Ghana (Korle Bu Teaching Hospital in Accra, and Komfo Anokye Teaching Hospital, and Peace and Love Hospital in Kumasi); or women presenting for treatment of pathologically-confirmed breast cancer at Korle Bu, Komfo Anokye, or Peace and Love Hospitals within one year of diagnosis. Controls were defined as women who had never been diagnosed with breast cancer. Controls were to be frequency-matched to cases based on area of residence, thus, we used hospital data from 2010–2012 to identify the geographic areas with the greatest number of breast cancer patients. The areas where it would be most feasible for potential controls to travel to the hospitals for study participation were selected for inclusion. The study area consisted of 22 municipal districts and metropolitan areas in Greater Accra, Central, and Eastern (i.e., Accra area) and Ashanti (i.e., Kumasi area) regions (Table 1). Control and case recruitment was limited to women residing in these areas.

**Table 1:** Areas included in the ghana breast health study household enumeration.

District *(sub-metros shown in italics)*	District/sub-metro code	Total number of EAs^a^	Number of EAs enumerated
**Greater Accra Region**			
Accra Metropolitan	0304		
*Ablekuma South*	*1*	304	2
*Ablekuma Central*	*2*	323	2
*Ashiedu Keteke*	*3*	152	2
*Osu Klotey*	*4*	119	2
*La*	*5*	207	2
*Ayawaso East*	*6*	195	2
*Ayawaso Central*	*7*	162	2
*Okai Koi South*	*8*	135	2
*Ablekuma North*	*9*	235	2
*Okai Koi North*	*10*	233	2
*Ayawaso West Wogon*	*11*	71	2
Adenta	0305	261	2
Ashaiman	0307	260	2
Dangbe West	0309	192	1
Dangbe East	0310	190	2
Ga East	0303	449	2
Ga West	0302	430	3
Weija (Ga South)	0301	699	3
Ledzokuku/Krowor	0306	288	2
Tema	0308		
*Tema West*	*1*	164	2
*Tema East*	*2*	200	2
*Kpone Katamanso*	*3*	154	2
**Central Region**			
Awutu Senya	0209	278	2
**Eastern Region**			
Akwapim South	0505	217	2
Suhum/Kraboa Coaltar	0504	298	2
**Ashanti Region**			
Kumasi Metropolitan	0614		
*Kwadaso*	*01*	278	5
*Nhyiaeso*	*02*	156	3
*Subin*	*03*	245	4
*Asokwa(Atonso)*	*04*	159	3
*Oforikrom*	*05*	287	9
*Asawase*	*06*	290	5
*Manhyia*	*07*	224	3
*Old Tafo*	*08*	231	3
*Suame*	*09*	152	3
*Bantama*	*10*	236	3
Atwima Kwanwoma	0613	152	3
Atwima Nwabiagya	0615	245	3
Bosumtwi	0612	174	3
Ejisu Juaben	0611	239	3
Afigya Kwabre	0619	252	4
Kwabre East	0620	193	2
Mampong	0622	164	2
Bekwai	0607	236	2

^a^EA–enumeration area

### Enumeration

Each of Ghana’s administrative regions is subdivided into districts or metropolitan areas; metropolitan areas are divided into sub-metros. Districts and sub-metros are further divided into enumeration areas—geographic subdivisions similar to a census tract with boundaries defined by the Ghana Statistical Service. District, sub-metro, and enumeration area definitions used in this study were based on definitions provided by the Ghana Statistical Service for the 2010 Population and Housing Census.

The first enumeration was conducted in November and December of 2012 by staff with extensive household enumeration experience and who previously worked on Ghana’s Population and Housing Census. A second enumeration was conducted in April 2014. At that time, the study catchment area was expanded to include Bekwai and Mampong municipal districts in the Ashanti region in order to capture potential cases residing in those districts. For each district or sub-metro, the enumeration areas selected for inclusion in this study were selected randomly, under the assumption that after the study eligibility criteria were applied, the eligible population in the enumeration area would be representative of the eligible population in the district/sub-metro as a whole. The number of enumeration areas randomly selected from each district or sub-metro for each enumeration was dictated by the district’s population density and the expected number of cases. We calculated the number of controls needed to match the expected number of cases within each age and district/sub-metro stratum and adjusted for an estimated non-participation rate to identify the number of controls we needed to approach for recruitment. The number of enumeration areas selected per district or sub-metro was then determined by ensuring that the enumerated population contained enough potential participants to approach in each age stratum.

Maps marking enumeration area boundary lines and landmarks were provided to the enumerators by the Ghana Statistical Service and information was collected using a standardized form. All known residences and households within an enumeration area were approached, including free-standing homes, households within large buildings and compound houses. Within compound houses, where several individuals or families live in distinct households sharing a common courtyard and cooking facilities, the unit of enumeration was the household, not the compound. Staff recorded the household location and the sex, age, name, and telephone number of each household member. If no one was available at the household, information was obtained from a neighbor or repeat visits were made (up to five contact attempts). In addition, enumerators informed residents of the purpose of the GBHS and the possibility of re-contact should they be chosen for the study. A brochure containing a description of the study and contact information for the principal investigators was left with each household. Enumeration records for women aged 18 to 74 years were compiled into a dataset for control selection.

### Control selection and recruitment

Systematic random sampling was used to select potential controls from the enumeration listings. Controls were frequency-matched to the expected age (in five-year age groups) and district of residence distributions of cases at a 1:1 ratio, where the expected age and district distributions and expected total number of cases were based on characteristics of Korle Bu, Komfo Anokye, and Peace and Love Hospitals’ breast cancer patients from 2010–2012. Potential controls sampled from the first enumeration dataset were approached for recruitment between February and September of 2013. Potential controls sampled from the second enumeration dataset were approached for recruitment from June 2014 to September of 2015. In Accra-area enumeration areas, potential controls were contacted by the staff who performed the enumeration. In the Kumasi-area enumeration areas, controls were contacted by study interviewers from Komfo Anokye and Peace and Love Hospitals. The first contact was made in-person when possible; however, some women were contacted by phone to determine a time to meet in person. At least five attempts were made to contact a potential control before it was determined that she could not be located.

We used several approaches to increase the likelihood of participation. In addition to distributing study brochures during the enumeration, we held discussions with community and church leaders and publicized the study through local media outlets to increase awareness of the study. Refusal conversion was attempted at the end of each control recruitment effort for a subset of women who initially stated they were not interested in the study or did not have time. These women were re-approached at least four months after the initial contact, and the study purpose and importance of their participation was explained again. Refusal conversion was carried out by a different interviewer than the one who made the initial request for participation. To maximize participation among this group of women for whom motivation and interest was low, we offered to allow completion of the interview-based questionnaire, saliva collection, blood draw, and waist and hip measurements at a private location other than the hospital (e.g., in the home or in a community center). As in the initial recruitment attempt, we made up to five attempts to contact a woman about the study; however, once the study interviewer made contact refusal conversion was only attempted once, after which a refusal was considered final.

Eligibility (described above, in Study Design) was determined based on an individual’s characteristics on the date that the study eligibility screener form was administered. An appointment was made to complete the study interview-based questionnaire after eligibility was confirmed.

### Data collection

Written informed consent was obtained prior to participation. All aspects of data collection were governed by detailed, written protocols that were disseminated to study staff at all three study hospitals and discussed in detail during interviewer trainings. Study questionnaire design, testing, and administration was described in detail previously [14]. Briefly, study participants were asked to complete an in-person interview-based questionnaire administered by a trained study interviewer in English or Twi, addressing demographics, reproductive and medical histories, body size, family history of breast cancer, and lifestyle factors (S1 File). As was described in detail by Brinton et al. [14], the questionnaire was focus group-tested by Ghanaian women in the US and Ghana before being used in this study. Standardized guidance regarding the intent of each question, prompts, and ways to re-phrase a question if it was not understood by the participant were outlined in a written question-by-question guide that was given to each interviewer. Throughout the questionnaire, selected questions were designated as “critical questions.” Participants who were unwilling or unable to complete the entire questionnaire were offered the opportunity to complete only the critical questions. Weight, standing height, sitting height, waist circumference, and hip circumference were measured using standardized protocols by trained staff; scales were calibrated periodically using metal weight standards. Additionally, participants were asked to provide a 2 mL saliva sample and a 20 mL blood sample. We endeavored to administer all study interviews at one of the recruiting hospitals. To facilitate that, women were offered transportation to the hospital or reimbursement for transportation costs. Participants were also offered a snack during the interview. If women were unable to travel to the hospital, the interview was conducted according to study protocols and the written question-by-question guide at their home or in a private space in the community. Blood collection was not performed for controls who completed participation in a location other than one of the study hospitals.

### Statistical analysis

We examined our ability to engage potential participants at several different stages, by evaluating successful contact (the number of women we were able to evaluate for eligibility divided by the total number of women sampled from the enumeration dataset), eligibility (the number of women deemed eligible divided by the total number of women for whom eligibility was assessed), and participation among eligible subjects (the number of women who completed the study interview divided by the total number of eligible women). Overall recruitment was evaluated by calculating the proportion of controls who participated out of the total number of potential controls that we contacted or attempted to contact.

Participant characteristics were tabulated based on responses to the questionnaire. Proportions were calculated among responses with non-missing data. Summaries of continuous data are reported as the median and interquartile range (IQR). Associations between variables were estimated using the chi-square, Fisher’s exact, or Kruskal Wallis tests.

## Results

### Enumeration

**Fig 2:**
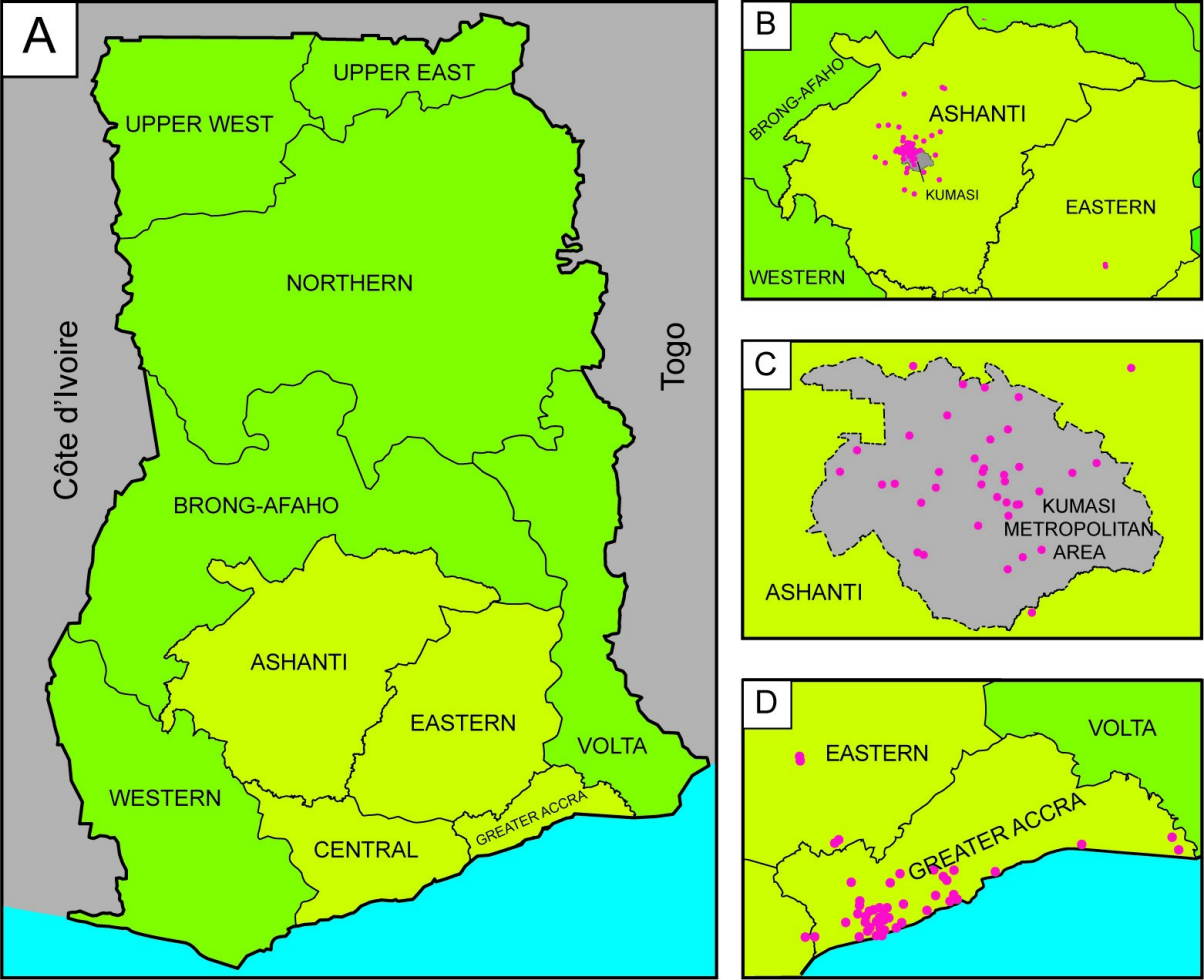
Areas selected for the recruitment of study controls in the ghana breast health study. Study control participants were recruited from Ghana’s Ashanti, Central, Eastern, and Greater Accra regions (highlighted in yellow, panel A). Each area is shown in greater detail in panels B, C, and D. The geographic locations of the enumeration areas used to identify the pool of potential controls are marked with pink dots. Within the Ashanti region, a large number of the enumeration areas were located within the Kumasi Metropolitan Area; a close-up view is shown in panel C.

One hundred and fourteen enumeration areas in 22 districts of the Greater Accra, Central, Eastern, and Ashanti regions were enumerated during the GHBS (Table 1 and Fig 2). There were no households for which basic demographic information could not be obtained from either a resident or a neighbor. One of the selected enumeration areas was near a university and most of its residents were students; therefore, a neighboring enumeration area was selected as a replacement. The enumeration resulted in a list of 11,171 women to be used as the control sampling frame for the case-control study: 5,622 women in the Accra area and 5,549 women in the Kumasi area.

### Recruitment

**Fig 3:**
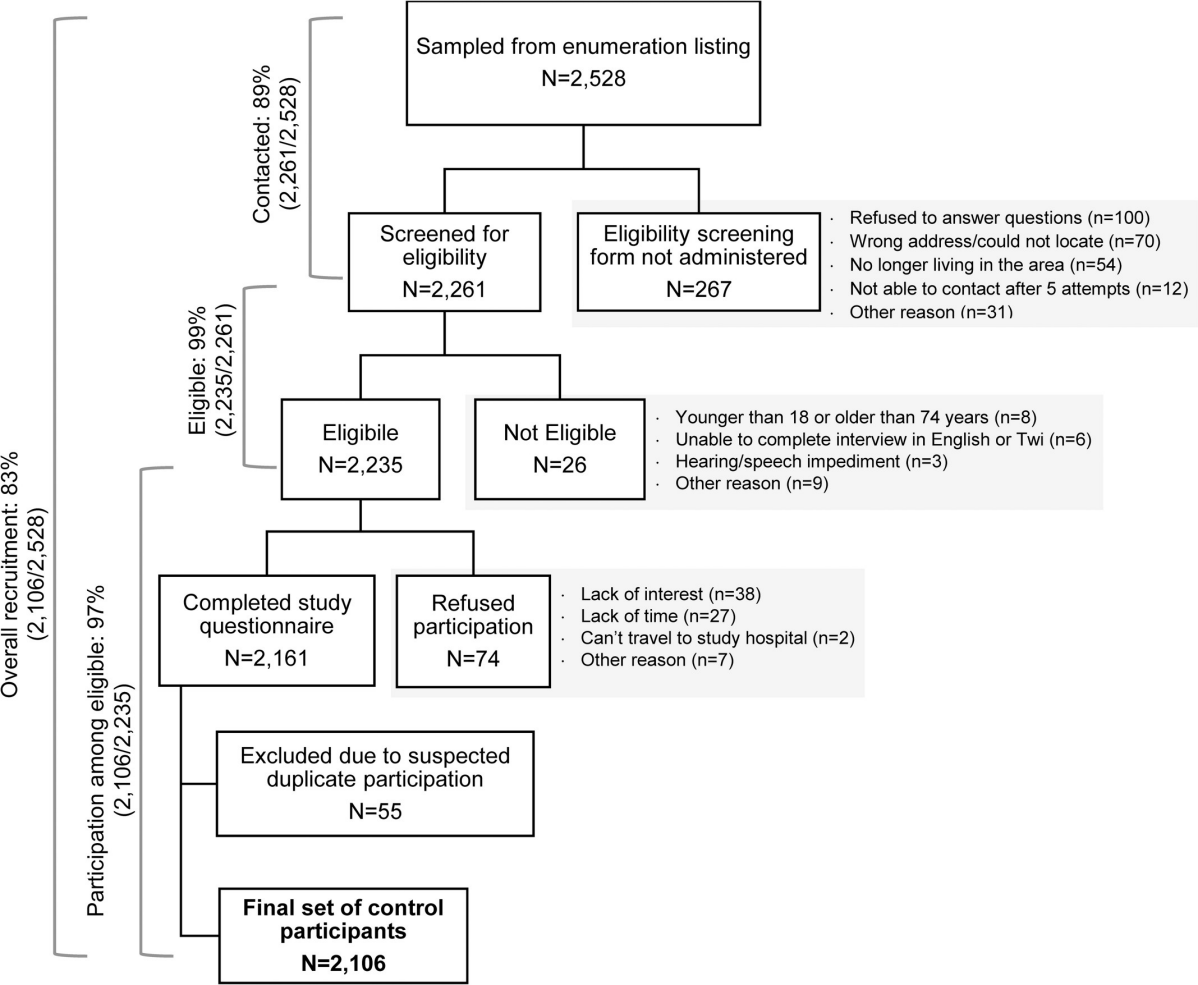
Flow chart of Ghana Breast Health Study control participation, 2013–2015, showing reasons for non-participation at right. Eligibility was based on self-reported demographic and health information and assessed using a standardized form. Participation was defined by completion of the study’s interview-based risk factor questionnaire. The final number of 2,106 participants includes 170 who were recruited using refusal conversion.

A total of 2,528 women were sampled from the enumeration dataset and approached for participation in the study (Fig 3). Study staff were able to contact and administer the eligibility screener to 2,261 women (89% of those approached). Our success in contacting potential controls was slightly higher in the Kumasi area (91%) than the Accra area (88%, P = 0.02). Additionally, women who were successfully contacted were older than women who could not be contacted (median age: 45 years *vs*. 42 years, respectively; P<0.01). The most common reasons that the eligibility form was not administered were that the woman refused to answer questions related to eligibility (37.5%), could not be located (26%), or no longer lived in the area (20%) (Fig 3).

Of the 2,261 women for whom eligibility was formally assessed, 2,235 met the eligibility criteria (99%). The most common reason for ineligibility was being outside the required age range of 18 to 74 years old (n = 8; Fig 3). Field notes from the recruiting staff suggest that the true proportion of eligible women may be slightly lower than what was detected throug formal eligibility assessments: a small number of women for whom the eligibility screener form was not completed were noted in study logs as having a comorbidity (n = 5), living outside the catchment area (n = 4), having a hearing or speaking problem (n = 1), being mentally incompetent (n = 1), or being outside the study age range (n = 1).

A total of 2,161 eligible women completed the risk factor questionnaire (97%). Participation among eligible subjects was higher in the Accra area than the Kumasi area (100% vs. 95%, respectively, P<0.01), but did not differ by age (P = 0.54). Among the women who refused to participate (including those who refused to answer questions regarding eligibility), the most common reasons for refusal to participate were lack of interest (61%) and lack of time (25%). After participation was completed, genetic data quality control checks indicated that 55 controls were duplicate observations, suggesting that some individuals were included in the control group multiple times. These samples were excluded from the study, resulting in a final group of 2,106 control participants. Thus, we recruited 83% of the women initially selected as potential controls.

### Characteristics of GBHS controls

The majority of GBHS controls were younger than 55 years old, married, and had junior secondary school education or higher (Table 2). Women recruited in the Accra area were most commonly born in Greater Accra (42%) or Eastern (19%) regions, whereas women recruited in the Kumasi area were most commonly born in the Ashanti region (82%). Eighty-six percent of study interviews were completed in study hospitals; other interviews were completed at the participant’s home or at a private location within the community (Table 3). Four percent of participants completed only the ‘critical questions’ or only part of the questionnaire (not restricted to critical questions) (median 30 minutes, IQR: 20–41 minutes). The proportion of women who completed the full questionnaire was not affected by whether the interview was conducted in the study hospital or in a community location (Table 3).

**Table 2:** Characteristics of ghana breast health study control participants, overall and according to recruitment method.

Variable	Overall		Recruitment method				
	N = 2,106		Refusal Conversion (N = 170)		First Approach (N = 1936)		P-value^a^
	N	%	N	%	N	%	
Age at interview (years)							
<35	435	21	49	29	386	20	0.04
35–44	561	27	44	26	517	27	
45–54	554	26	42	25	512	27	
≥55	546	26	35	21	511	27	
Unknown/missing	10		0		10		
Marital status							
Married	1112	62	83	64	1029	62	0.02
Living with partner	134	7	14	11	120	7	
Single/never married	249	14	8	6	241	15	
Widowed	222	12	15	12	207	12	
Divorced/separated	71	4	9	7	62	4	
Unknown/missing	318		41		277		
Highest level of education							
No formal education	498	24	35	23	463	25	0.03
Primary school	369	18	18	12	351	19	
Junior secondary	647	32	68	44	579	31	
Senior secondary	283	14	16	10	267	14	
Some college, vocational, or technical	136	7	8	5	128	7	
Completed college or higher	43	2	3	2	40	2	
Other	62	3	5	3	57	3	
Unknown/missing	68		17		51		
Region of birth							
*Overall*							
Ashanti	1138	56	63	41	1075	57	<0.01
Greater Accra	312	15	32	21	280	15	
Eastern	165	8	15	10	150	8	
Volta	107	5	11	7	96	5	
Other region or outside Ghana	316	16	32	21	284	15	
Unknown/missing	68		17		51		
*Recruited in Accra area*							
Ashanti	41	6	6	7	35	6	0.43
Greater Accra	288	42	31	36	257	42	
Eastern	134	19	15	17	119	20	
Volta	88	13	10	12	78	13	
Other region or outside Ghana	142	20	24	28	118	20	
Unknown/missing	43		17		26		
*Recruited in Kumasi area*							
Ashanti	1097	82	57	85	1040	81	0.81
Greater Accra	24	2	1	1	23	2	
Eastern	31	2	0	0	31	2	
Volta	19	1	1	1	18	1	
Other region or outside Ghana	174	13	8	12	166	13	
Unknown/missing	25		0		25		
Age at first menstrual period (years)							
<15	568	30	59	37	509	29	0.12
15	548	29	36	23	512	30	
16	383	20	34	21	349	20	
≥17	395	21	30	19	365	21	
Unknown/missing	212		11		201		
Parity							
Nulliparous	228	11	19	12	209	11	<0.01
1–2	533	25	59	36	474	25	
3–4	685	33	44	27	641	33	
≥ 5	652	31	42	26	610	32	
Unknown/missing	8		6		2		
Menopausal status							
Premenopausal	1218	58	114	67	1104	58	0.01
Postmenopausal	870	42	55	33	815	42	
Unknown/missing	18		1		17		
First-degree family history of breast cancer							
No	2036	98	162	99	1874	98	0.37
Yes	46	2	2	1	44	2	
Unknown/missing	24		6		18		

^a^P-values were calculated using the chi-square test, with the exception of region of birth among women recruited in the Kumasi area, which was calculated using the Fisher exact test.

**Table 3:** Ghana Breast Health Study interview-based questionnaire completion among 2,106 controls, 2013–2015.

			*Interview Location*^*a*^			
Questionnaire completion status	All controls		Hospital		Home/other location	
	N	%	N	%	N	%
Full questionnaire	2030	96	1749	96	279	96
Critical questions or partial questionnaire	76	4	63	4	12	4
Total	2106		1812		291	

^a^Interview location was missing for 3 subjects

### Characteristics of controls enrolled using refusal conversion

We approached 299 women who initially expressed a lack of time or interest, or who were away from home at the time of the initial recruitment visit, using our refusal conversion protocol. Women who were too ill to participate or otherwise ineligible were not approached for refusal conversion. In total, 170 participants were ultimately enrolled through refusal conversion. Twenty-three percent of the refusal conversion enrollees completed their interview in a community location rather than at one of the designated study hospitals. In addition, women who were enrolled using refusal conversion were more often younger, premenopausal, married or living with a partner, and had fewer children when compared with women who participated when first approached (Table 2).

## Discussion

A collaboration between the National Cancer Institute and three major hospitals in Ghana, the GBHS is a case-control study that aims to examine risk associations and estimate the population prevalence of known and novel breast cancer risk factors in a West African population. We prospectively identified a pool of population controls within a defined geographic area prior to the commencement of the study. Our two-stage sampling methodology (sampling enumeration areas and sampling potential controls within enumeration areas) was based in part on a study of benign prostatic hyperplasia and lower urinary tract symptoms conducted previously in Accra that used a three-stage sampling design to select participants [15]. Our generation of a pool of controls prior to the study’s start, rather than recruiting controls after cases have been recruited, meant that control and case groups completed their study participation during the same time period, preventing potential bias that may have occurred if the two groups were interviewed during different time periods. Population-based sampling has been used previously in Ghana for the conduct of health surveys [15–17] and in other parts of Africa to survey health status and chronic disease risk factors, including a case-control study of Burkitt lymphoma [18–25]. The only breast cancer study conducted in sub-Saharan Africa with a similar recruitment approach is the Nigerian Breast Cancer Study, which recruited population controls from a single community [13]. Overall, 83% of potential controls we contacted or attempted to contact participated in the GBHS, including 94% of eligible women. The Nigerian Breast Cancer Study reported similarly high (98%) participation among eligible controls [13]. Our experience demonstrates that population-based research can be conducted in Africa with relatively little loss of the eligible population due to non-participation.

Aspects of our approach were developed based on consultation with medical professionals in Ghana and from data published by the Nigerian study [13]. Both sources emphasized the importance of engaging community and church leaders prior to recruitment, which likely contributed to the high participation. We also gained insight from a pilot study conducted in Nigeria and Kenya [26], in which questionnaire length and lack of transportation were barriers to participation. Thus, we drafted several iterations of our questionnaire and tested it using focus groups prior to initiating the case-control study to ensure that the length would be acceptable. We also provided transportation, reimbursed participants for transportation, or as a last resort, conducted the interview in the field. Additionally, the study brochure provided during the enumeration process answered questions regarding the purpose of the study prior to the recruitment attempt. Interviewers were advised that the first study contact should be in-person, when possible, so that additional questions could be addressed immediately. We believe that these measures contributed to the success of the recruitment.

The most common reasons for loss of potential controls were a refusal to participate and non-contact. The number of women cited as ineligible and/or refusing at each step of the study differ slightly from what was reported in Brinton et al. [14] due to the way we have described the eligibility screening aspect of the recruitment process. Our refusal conversion protocol was successful in decreasing the number of refusals—more than 50% of women approached during refusal conversion were ultimately enrolled in the study. The main difference between the initial invitation and refusal conversion invitation to participate in the study was the ability to complete the interview at home or in a community location, suggesting that this flexibility was a valuable incentive. We excluded responses from 55 participants that appeared to have duplicated their participation. This resulted in a final number of enrolled participants of 2,106, rather than the 2,161 initially cited in Brinton et al. [14]. These duplicate participants were discovered through a genetic data “identifiler” analysis and demonstrate the importance of conducting such data checks, when possible.

The GBHS used two approaches for contacting controls, both with advantages and disadvantages. In Accra, the enumeration staff initiated the recruitment process. Although the staff were familiar with the households from the enumeration, there was a high initial refusal rate which we suspect was due to the fact that the enumeration staff was male. Accordingly, female interviewers conducted the Accra-area refusal conversion and were successful in getting two-thirds of initial refusals to participate. In contrast, in Kumasi the recruitment process was initiated by female interviewers who were not involved in the enumeration. The interviewers reported difficulty locating the potential controls, including some instances where the name recorded during the enumeration was not the name the woman was commonly known by in the community. Our experience indicated that use of female recruiters and the knowledge of the enumeration team were both essential to successful recruitment. To lessen these issues after the first enumeration and recruitment effort, procedures were modified so that paired teams of interviewers and enumeration staff recruited controls together. In future studies, the inclusion of women on the enumeration team may also help reduce refusals.

The GBHS was successful in convincing a majority of controls to come to the hospital to complete the study interview. We wanted controls to come to the hospital for four reasons: i) so that control interviews would be completed in an environment comparable to case interviews; ii) to provide privacy for questionnaire completion; iii) so that there would be qualified personnel on site in case there were complications related to the blood draw; and iv) to decrease the time between biospecimen collection and processing. The number of women whose participation was gained only by administering the questionnaire in the field was small. Although the setting of home and community interviews varied, the relative frequency of interviews conducted away from the study hospitals was low. Steps were taken to ensure that the conduct of the interview was the same regardless of the location. These steps included the use of a written protocol and question-by question guide that was used by each interviewer. We acknowledge that it is possible that study participants may have answered questions differently depending on whether or not they were in a hospital setting. However, we believe that the measures we put in place to standardize the process greatly reduced those chances and that inclusion of these controls in the study reduces the chance of selection bias due to differential non-participation. In future studies, we will conduct sensitivity analyses to determine whether results are influenced by location of study completion.

Though our approach to identifying and recruiting controls resulted in high levels of participation, there were limitations. In theory, population controls should represent the population from which the study cases arise. However, it is difficult to evaluate comparability because we lack complete knowledge of what determines whether a woman who suspected she had breast cancer would go to one of the hospitals included in this study, as opposed to a smaller clinic, a traditional healer, or not seeking any care. Limitations related to unknown patterns of treatment-seeking behaviors would exist regardless of the control population we selected. For example, if hospital controls had been selected we could not be sure that the selection pressures that caused them to seek care at the hospital were the same as the pressures that motivated breast cancer cases to seek care [27]. Another limitation was the labor-intensive nature of the enumeration process, which limited the number of enumerations that could be performed. We maximized the efficiency of the process by employing staff who had extensive experience with the process. Still, it is possible that the length of time between the enumeration and when study staff attempted to contact a woman for recruitment may have contributed to the inability to contact some women. Finally, there was variation in the location and the language in which women completed the interview-based questionnaire. It is possible that these differences may have influenced how women answered the interview questions.

## Conclusions

In summary, we successfully recruited population controls in a breast cancer case-control study in Ghana. By enumerating a random subset of the population within the study catchment area, we were able to identify a set of controls that were representative of the study population base, conditional on the control matching factors of district of residence and age. Our experience demonstrates that traditional, population-based epidemiologic methods can be employed in sub-Saharan Africa. The techniques we used may work in other countries with similar infrastructure challenges to increase epidemiologic knowledge of health in underserved populations.

## Supporting information

S1 File Ghana breast health study questionnaire. (PDF)

S1 Table Ghana breast health study recruiting population controls minimal dataset. (XLSX)
