# Supplementary material for: Recruiting population controls for case-control studies in sub-Saharan Africa: The Ghana Breast Health Study
Source: PLoS One. 2019 Apr 16;14(4):e0215347. doi: 10.1371/journal.pone.0215347 (PMC6467449; doi:10.1371/journal.pone.0215347)
Supplement: S1 File — (PDF) [file pone.0215347.s001.pdf]

Participant Barcode:

Ghana Breast Health Study

Risk Factor Questionnaire Version 2

★**DATE OF INTERVIEW:** |__|__| / |__|__|__| / | 2 | 0 |__|__|
DAY MONTH* YEAR
*(*Record in 3-letter abbreviations, e.g., JAN)*

★**INTERVIEWER'S ID:** |__|__|__|__|__|__|
Please print ID clearly

★**STUDY MANAGER'S ID:** |__|__|__|__|__|__|
Please print ID clearly

★**STUDY SITE:** |__|__|
01 Korle Bu Teaching Hospital, Accra, Ghana (KBTH)
02 Komfo Anokye Teaching Hospital, Kumasi, Ghana (KATH)
03 Peace and Love Hospital, Kumasi, Ghana (PLH)

★**INTERVIEW START TIME:** |__|__| : |__|__| Use 24 hr clock. Example: 14:00

THIS PAGE INTENTIONALLY BLANK

Introduction: Thank you for agreeing to participate in our study. Today, I would like to ask you some questions related to your lifestyle and your health. All of the answers you give me will be kept confidential and your name will never be connected to your answers in any way. I want to remind you that your participation is voluntary. Although it is very important we obtain answers from each and every participant, you do not have to answer any questions you do not wish to answer.

SECTION A. BACKGROUND INFORMATION

First I would like to ask for some background information about you and your family.

★A1. What is your date of birth? (RECORD MONTH IN 3-LETTER ABBREVIATIONS LIKE JAN, FEB, MAR,…DEC. IF UNKNOWN, ENTER 99 FOR DAY, XXX FOR MONTH, 9999 FOR YEAR.)

|__|__| / |__|__|__| / |__|__|__|__|
DAY MONTH YEAR

★A2. What is your current age? (IF UNKNOWN, ENTER 99.)

|__|__|
AGE

A3. Which of the following best describes your current marital status? (CHOOSE ONE.)

Married.. 1
Living with a partner 2
Widowed.. 3
Divorced.. 4
Separated.. 5
Single/Never Married............................. 6

A4. What is the highest level of education you have completed? (CHOOSE ONE.)

SHOW CARD A4

NO FORMAL EDUCATION/SOME PRIMARY SCHOOL.............. 01
PRIMARY SCHOOL.. 02
JUNIOR SECONDARY SCHOOL (JSS) 03
SENIOR SECONDARY SCHOOL (SS).. 04
6TH FORM 05
VOCATIONAL/TECHNICAL/POLYTECHNIC/SOME COLLEGE . 06
COMPLETED DEGREE AT COLLEGE/UNIVERSITY.................. 07
MASTERS DEGREE 08
PHD, MD, JD, OTHER TERMINAL PROFESSIONAL DEGREE.. 09
OTHER (SPECIFY) 10
____
DON'T KNOW/DON'T REMEMBER.. 99

A5. What religion do you currently practice? (CHOOSE ONE.)

CHRISTIANITY	1
MUSLIM/ISLAM	2
TRADITIONALIST	3
OTHER (SPECIFY) ________________	4
NO RELIGION	5

A6. In what region were you born? (CHOOSE ONE.)

SHOW CARD A6

ASHANTI	01
BRONG AHAFO	02
CENTRAL	03
EASTERN	04
GREATER ACCRA	05
NORTHERN	06
UPPER WEST	07
UPPER EAST	08
VOLTA	09
WESTERN	10
OUTSIDE GHANA (SPECIFY COUNTRY) ________________	11

A7. In what region have you lived for most of your life? (CHOOSE ONE.)

SHOW CARD A7

ASHANTI	01
BRONG AHAFO	02
CENTRAL	03
EASTERN	04
GREATER ACCRA	05
NORTHERN	06
UPPER WEST	07
UPPER EAST	08
VOLTA	09
WESTERN	10
OUTSIDE GHANA (SPECIFY COUNTRY) ________________	11

A8. To which ethnic groups do you belong? (SELECT ALL THAT APPLY.)

SHOW CARD A8		
	AKAN	01
	GA-DANGME	02
	EWE	03
	GUAN	04
	GURMA	05
	MOLE-DAGBANI	06
	GRUSI	07
	MANDE	08
	OTHER ETHNIC GROUP (SPECIFY) ______________________	09

Now I will ask you some questions about your home. When answering these questions, please consider your home as the place you live primarily with your immediate family; that is those family members with whom you share everyday meals and possibly share the same sleeping area. Please do not include extended family members that may live in the same building with you.

A9. Does your home have electricity? (Do not include power from home generator.)

YES	1
NO	2
DON'T KNOW	9

A10. Do you or does anyone in your home own the following items?

a. A mobile telephone

YES	1
NO	2
DON'T KNOW	9

b. A television

YES	1
NO	2
DON'T KNOW	9

c. A car or truck

YES	1
NO	2
DON'T KNOW	9

d. A refrigerator

YES	1
NO	2
DON'T KNOW	9

e. A computer

YES	1
NO	2
DON'T KNOW	9

A11. What is the main type of flooring in your home?

DIRT	1
CEMENT	2
CERAMIC TILE	3
TERRAZO	4
WOOL CARPET	5
OTHER (SPECIFY) ________________	6
DON'T KNOW	9

A12. What is the main type or style of window in your home?

WOOD	1
LEVERS	2
TINTED	3
OTHER (SPECIFY) ________________	4

SECTION B. PREGNANCY HISTORY

Now I'd like to ask you some questions about your pregnancy history. Please think about all births, stillbirths, miscarriages, abortions, tubal pregnancies and ectopic pregnancies you have experienced.

★**B1.** **How many pregnancies have you had? If you are currently pregnant, include this pregnancy in your count. If you were pregnant with multiples, count this as one pregnancy. (IF UNKNOWN, ENTER 99. IF NONE, ENTER 00.)**

|__|__|
PREGNANCIES

IF ANSWER TO B1 IS UNKNOWN OR NONE, THEN GO TO B7.

IF ANSWERING ONLY ★ CRITICAL QUESTIONS, THEN GO TO B9 AND B10 AT END OF SECTION B.

Now I'm going to ask you some questions about each of your ___ pregnancies. IF RESPONDENT IS CURRENTLY PREGNANT, RECORD IT AS THE 1ST PREGNANCY. IF RESPONDENT IS NOT CURRENTLY PREGNANT, START BY ASKING THE RESPONDENT ABOUT HER FIRST PREGNANCY. ASK ALL QUESTIONS ABOUT EACH PREGNANCY BEFORE MOVING ONTO THE NEXT PREGNANCY. AFTER ALL PREGNANCY INFORMATION HAS BEEN RECORDED, MOVE TO SECTION C.

THE GRID SHOULD BE COMPLETED BY ASKING QUESTIONS DOWN EACH COLUMN BEFORE MOVING ON TO THE NEXT COLUMN.

B2. Did this pregnancy end as a live birth, stillbirth, miscarriage, abortion, tubal pregnancy, or ectopic pregnancy?

<table>
<tr><th>a. PREGNANCY</th><th>b. PREGNANCY</th><th>c. PREGNANCY</th></tr>
<tr>
<td>CURRENTLY PREGNANT
.... 1
(GO TO NEXT PREGNANCY)
LIVE BIRTH...................... 2
MULTIPLE BIRTHS 3
STILLBIRTH
(≥5 MONTHS) 4
MISCARRIAGE
(<5 MONTHS) 5
TUBAL OR ECTOPIC
PREGNANCY 6
INDUCED ABORTION 7
OTHER (SPECIFY).......... 8
____________________</td>
<td>LIVE BIRTH..................... 2
MULTIPLE BIRTHS......... 3
STILLBIRTH
(≥5 MONTHS).............. 4
MISCARRIAGE
(<5 MONTHS).............. 5
TUBAL OR ECTOPIC
PREGNANCY............... 6
INDUCED ABORTION 7
OTHER (SPECIFY) 8
____________________</td>
<td>LIVE BIRTH 2
MULTIPLE BIRTHS 3
STILLBIRTH
(≥5 MONTHS) 4
MISCARRIAGE
(<5 MONTHS) 5
TUBAL OR ECTOPIC
PREGNANCY 6
INDUCED ABORTION..... 7
OTHER (SPECIFY).......... 8
____________________</td>
</tr>
</table>

B3. Please tell me when your pregnancy ended or your baby was born. (RECORD RESPONDENT'S AGE ESTIMATE IF SHE CANNOT REMEMBER MONTH AND YEAR.)

<table>
<tr>
<td>|__|__|__| / |__|__|__|__|
MONTH YEAR

MOTHER'S AGE
ESTIMATE _______</td>
<td>|__|__|__| / |__|__|__|__|
MONTH YEAR

MOTHER'S AGE
ESTIMATE _______</td>
<td>|__|__|__| / |__|__|__|__|
MONTH YEAR

MOTHER'S AGE
ESTIMATE _______</td>
</tr>
</table>

B4. ONLY IF MULTIPLE BIRTHS: How many live and still birth babies resulted from this pregnancy?

<table>
<tr>
<td>LIVE BIRTHS: |__|__|

STILL BIRTHS: |__|__|</td>
<td>LIVE BIRTHS: |__|__|

STILL BIRTHS: |__|__|</td>
<td>LIVE BIRTHS: |__|__|

STILL BIRTHS: |__|__|</td>
</tr>
</table>

B5. ONLY IF LIVE BIRTH: Did you breastfeed this baby/these babies?

<table>
<tr>
<td>YES................................. 1
NO................................... 2
(IF NO, GO TO NEXT
PREGNANCY)</td>
<td>YES 1
NO 2
(IF NO, GO TO NEXT
PREGNANCY)</td>
<td>YES................................. 1
NO 2
(IF NO, GO TO NEXT
PREGNANCY)</td>
</tr>
</table>

B6. ONLY IF BREAST FED: How long did you breastfeed this baby/these babies? (IF LESS THAN ONE MONTH, CODE AS "0" MONTHS.)

<table>
<tr>
<td>|__|__|__|
MONTHS</td>
<td>|__|__|__|
MONTHS</td>
<td>|__|__|__|
MONTHS</td>
</tr>
</table>

B2. Did this pregnancy end as a live birth, stillbirth, miscarriage, abortion, tubal pregnancy, or ectopic pregnancy?

d. PREGNANCY	e. PREGNANCY	f. PREGNANCY
LIVE BIRTH...................... 2<br>MULTIPLE BIRTHS .... 3<br>STILLBIRTH<br>(≥5 MONTHS) .... 4<br>MISCARRIAGE<br>(<5 MONTHS) .... 5<br>TUBAL OR ECTOPIC<br>PREGNANCY .... 6<br>INDUCED ABORTION ..... 7<br>OTHER (SPECIFY).......... 8<br>______________________	LIVE BIRTH..................... 2<br>MULTIPLE BIRTHS......... 3<br>STILLBIRTH<br>(≥5 MONTHS).............. 4<br>MISCARRIAGE<br>(<5 MONTHS).............. 5<br>TUBAL OR ECTOPIC<br>PREGNANCY............... 6<br>INDUCED ABORTION .... 7<br>OTHER (SPECIFY) .... 8<br>______________________	LIVE BIRTH .... 2<br>MULTIPLE BIRTHS .... 3<br>STILLBIRTH<br>(≥5 MONTHS) .... 4<br>MISCARRIAGE<br>(<5 MONTHS) .... 5<br>TUBAL OR ECTOPIC<br>PREGNANCY .... 6<br>INDUCED ABORTION..... 7<br>OTHER (SPECIFY).......... 8<br>______________________

B3. Please tell me when your pregnancy ended or your baby was born. (RECORD RESPONDENT'S AGE ESTIMATE IF SHE CANNOT REMEMBER MONTH AND YEAR.)

|__|__|__| / |__|__|__|__| MONTH YEAR<br>MOTHER'S AGE<br>ESTIMATE _______	|__|__|__| / |__|__|__|__| MONTH YEAR<br>MOTHER'S AGE<br>ESTIMATE _______	|__|__|__| / |__|__|__|__| MONTH YEAR<br>MOTHER'S AGE<br>ESTIMATE _______

B4. ONLY IF MULTIPLE BIRTHS: How many live and still birth babies resulted from this pregnancy?

LIVE BIRTHS: |__|__| STILL BIRTHS: |__|__|	LIVE BIRTHS: |__|__| STILL BIRTHS: |__|__|	LIVE BIRTHS: |__|__| STILL BIRTHS: |__|__|

B5. ONLY IF LIVE BIRTH: Did you breastfeed this baby/these babies?

YES................................. 1<br>NO................................... 2<br>(IF NO, GO TO NEXT PREGNANCY)	YES .... 1<br>NO .... 2<br>(IF NO, GO TO NEXT PREGNANCY)	YES................................. 1<br>NO .... 2<br>(IF NO, GO TO NEXT PREGNANCY)

B6. ONLY IF BREAST FED: How long did you breastfeed this baby/these babies? (IF LESS THAN ONE MONTH, CODE AS "0" MONTHS.)

|__|__|__| MONTHS	|__|__|__| MONTHS	|__|__|__| MONTHS

IF MORE THAN 6 PREGNANCIES, USE ADDITIONAL PAGES TO RECORD DETAILS ABOUT EACH ADDITIONAL PREGNANCY (FOUND AT END OF THIS QUESTIONNAIRE).

B7. Have you ever encountered difficulty that lasted more than one year in getting pregnant?

YES..	1	
NO	2	(SECTION C)
DON'T KNOW	9	(SECTION C)

B8. Did you receive treatment in order to help you get pregnant?

YES..	1
NO	2
DON'T KNOW	9

CRITICAL QUESTIONS ONLY:

★B9. During what month and year did your first full-term pregnancy end? A full-term pregnancy is one that lasted 5 months or longer. Include stillbirths and live births.

|__|__|__| / |__|__|__|__|
MONTH YEAR

★B10. How many full-term pregnancies have you had?

|__|__| **(SECTION C)**
PREGNANCIES

SECTION C. MENSTRUAL HISTORY AND MENOPAUSE

Now I would like to ask you some details about your menstrual period.

★C1. At what age did you have your first menstrual period? (IF NEVER, ENTER 98 AND SKIP TO C6. IF UNKNOWN, ENTER 99.)

|__|__|
AGE

C2. Thinking about all the years when you had a menstrual period, could you usually predict when your menstrual period would start?

YES	1	
NO	2	
DON'T KNOW	9	

★C3. Are you still having menstrual periods? Please do not include temporary breaks due to pregnancy, breastfeeding or family planning.

**YES**	**1**	**(C6)**
**NO**	**2**	
**DON'T KNOW/NOT SURE**	**9**	**(C6)**

★C4. How old were you when your menstrual periods stopped completely? (IF UNKNOWN, ENTER 99.)

|__|__|
AGE

C5. Why did your menstrual period stop? (RECORD ONE.)

NATURAL MENOPAUSE (PERIOD STOPPED BY ITSELF)	1	
SURGERY	2	(C7)
RADIATION OR CHEMOTHERAPY	3	
MEDICATION OTHER THAN CHEMOTHERAPY (SPECIFY) ______	4	
OTHER (SPECIFY) ______	5	
DON'T KNOW	9	

C6. Have you ever had any surgery(ies) that resulted in the removal of both of your ovaries?

YES	1	(C8)
NO	2	(SECTION D)
DON'T KNOW	9	(SECTION D)

C7. Did the surgery result in the removal of both of your ovaries?

YES.. 1
NO .... 2 (SECTION D)
DON'T KNOW .... 9 (SECTION D)

C8. How old were you when you had the most recent surgery that resulted in the removal of both of your ovaries? (IF UNKNOWN, ENTER 99.)

|__|__|
AGE

SECTION D. FAMILY PLANNING

THE GRID SHOULD BE COMPLETED BY ASKING QUESTIONS ACROSS EACH ROW BEFORE MOVING ON TO THE NEXT ROW.

Now I'd like to ask you about birth control for family planning.

★**a. Have you ever used [bcm]?**	★**b. How old were you when you first started [bcm]? (IF UNKNOWN, ENTER 99.)**	★**c. Are you currently using [bcm]?**	d. How old were you when you last used [bcm]? (IF UNKNOWN, ENTER 99.)	e. In total, for how long did you use [bcm]? Do not include breaks when you were not using [bcm]. (IF UNKNOWN, ENTER 999.)
★**D1. Birth control pills?**<br>**YES ....1**<br>**NO ....2 (D2)**<br>**DON'T KNOW ..9 (D2)**	**|__|__|**<br>**AGE**	**YES........... 1 (e)**<br>**NO............ 2**	|__|__| AGE	|__|__|__| DAYS ...... 1<br>MONTHS 2<br>YEARS.... 3
★**D2. An implant placed under the skin every 5 years?**<br>**YES ....1**<br>**NO ....2 (D3)**<br>**DON'T KNOW ..9 (D3)**	**|__|__|**<br>**AGE**	**YES........... 1 (e)**<br>**NO............ 2**	|__|__| AGE	|__|__|__| DAYS ...... 1<br>MONTHS 2<br>YEARS.... 3
★**D3. An injection, given every 3 months?**<br>**YES ....1**<br>**NO ....2 (D4)**<br>**DON'T KNOW ..9 (D4)**	**|__|__|**<br>**AGE**	**YES........... 1 (e)**<br>**NO............ 2**	|__|__| AGE	|__|__|__| DAYS ...... 1<br>MONTHS 2<br>YEARS.... 3
★**D4. Any other hormonal method? (SPECIFY)**<br>________________<br>**YES ....1**<br>**NO ....2 (SECTION E)**<br>**DON'T KNOW ..9 (SECTION E)**	**|__|__|**<br>**AGE**	**YES........... 1 (e)**<br>**NO............ 2**	|__|__| AGE	|__|__|__| DAYS ...... 1<br>MONTHS 2<br>YEARS.... 3

SECTION E. HEIGHT AND WEIGHT

Now I'd like to ask you about your height and weight at different times during your life.

E1. At the time of your first menstrual period, were you…?

A lot shorter than other girls	1	
Somewhat shorter than other girls	2	
Same height as other girls......................	3	
Somewhat taller than other girls............	4	
A lot taller than other girls.......................	5	
Never had menstrual period	6	(E3)

E2. At the time of your first menstrual period, were you…?

A lot thinner than other girls....................	1
Somewhat thinner than other girls..........	2
Same weight as other girls	3
Somewhat heavier than other girls.........	4
A lot heavier than other girls..................	5

★E3. What is the tallest you have ever been without shoes? (IF UNKNOWN, ENTER 999.)

|__|__|__| OR |__|__| |__|__|
HEIGHT (CM) FEET INCHES

E4. How old were you when you reached that height? (IF UNKNOWN, ENTER 99.)

|__|__|
AGE

E5. How much did you weigh 5 years ago? (IF UNKNOWN, ENTER 999.)

|__|__|__| OR |__|__|__|
WEIGHT (KG) WEIGHT (LB)

★E6. Compared to your current weight, would you say your weight five years ago was:

**Much less than now ....**	**1**
**A little less than now...........................**	**2**
**About the same as now ....**	**3**
**A little more than now..........................**	**4**
**Much more than now...........................**	**5**

Please look at these silhouettes (SHOW CARD):

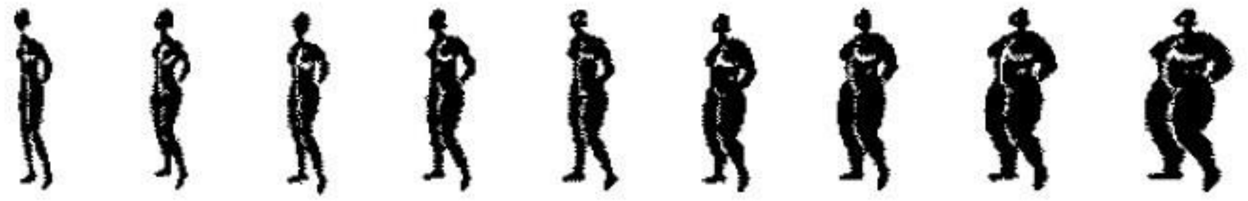

1 2 3 4 5 6 7 8 9

E7. Which silhouette best represents your body shape for **most** of your adult years (from the age of 18)?

SHOW CARD E7-E9	|__| SILHOUETTE

E8. Has your silhouette changed within the last five years?

SHOW CARD E7-E9		
	YES..	1
	NO	2 (E10)
	DON'T KNOW	9

E9. Which silhouette best represents your current body shape?

SHOW CARD E7-E9	|__| SILHOUETTE

★E10. As an adult, when you have gained weight, where on your body did you mainly tend to add the weight? (RECORD ONE ANSWER ONLY.)

**DON'T GAIN WEIGHT..**	**1**
**AROUND THE CHEST AND UPPER BODY ....**	**2**
**AROUND THE WAIST/STOMACH..........................**	**3**
**AROUND THE HIPS ....**	**4**
**EQUALLY ALL OVER ....**	**5**
**OTHER (SPECIFY) ....**	**6**

____

SECTION F. ALCOHOL USE

These next questions deal with a sensitive topic, but it is important for the study that you answer them truthfully. As a reminder, your answers are confidential. I will be asking you about drinking alcoholic beverages such as beer, wine, or hard liquor (gin).

★F1. Has there ever been a time in your life when you had at least one drink a month?

YES.. 1
NO .... 2 (SECTION G)
DON'T KNOW .... 9 (SECTION G)

★F2. For how long did you have at least one drink a month: Would you say less than six months, or six months or longer?

LESS THAN SIX MONTHS.................... 1 (SECTION G)
SIX MONTHS OR LONGER 2

To answer the next set of questions, I want you to think specifically about the times when you drank alcohol at least once a month for 6 months or longer.

★F3. How old were you when you first started drinking alcoholic beverages at least once a month? (IF UNKNOWN, ENTER 99.)

|__|__|
AGE

F4. When you drank, how often did you usually drink? You can tell me the number of times per day, week, or month. (IF UNKNOWN, ENTER 999.)

|__|__|__| PER: DAY........................ 1
TIMES WEEK..................... 2
MONTH 3

F5. How many drinks did you usually have on those days that you drank? (IF UNKNOWN, ENTER 99.)

|__|__|
DRINKS

★F6. Do you currently drink alcoholic beverages at least once a month?

YES... 1 (SECTION G)
NO 2
DON'T KNOW .... 9 (SECTION G)

F7. At what age did you stop drinking alcoholic beverages at least once a month? (IF UNKNOWN, ENTER 99.)

|__|__|
AGE

SECTION G. TOBACCO USE

These next questions deal with a sensitive topic, but it is important for the study that you answer them truthfully. As a reminder, your answers are confidential. I will ask you some questions about your smoking history.

G1. Altogether, have you smoked a total of 100 cigarettes or more in your lifetime?

YES..	1	
NO	2	(G8)
DON'T KNOW	9	(G8)

G2. Did you ever smoke at least one cigarette each day for six months or longer?

YES..	1	
NO	2	(G8)
DON'T KNOW	9	(G8)

G3. How old were you when you first started smoking cigarettes daily? (IF UNKNOWN, ENTER 99.)

|__|__|
AGE

G4. Do you currently smoke at least one cigarette per day?

YES..	1	(G6)
NO	2	

G5. How old were you when you last stopped smoking cigarettes daily? (IF UNKNOWN, ENTER 99.)

|__|__|
AGE

G6. In total, for how many years have you smoked cigarettes daily? Please do not count any years when you did not smoke cigarettes. (IF UNKNOWN, ENTER 99.)

|__|__|
YEARS

G7. Thinking about all the years when you smoked daily, how many cigarettes did you usually smoke in a day? (IF UNKNOWN, ENTER 999.)

|__|__|__|
CIGARETTES/DAY

G8. Have you ever used any form of smoked tobacco products other than cigarettes (for example, cigars, water pipe, cigarillos, little cigars, or pipes) at least once per day for six months or longer?

YES	1	
NO	2	
DON'T KNOW	9	

★G9. Have you ever used any form of smokeless tobacco products (for example, chewing tobacco, snuff, or dip) regularly, that is, at least once per day for six months or longer?

**YES**	**1**	
**NO**	**2**	**(SECTION H)**
**DON'T KNOW**	**9**	**(SECTION H)**

★G10. How old were you when you first started using smokeless tobacco products daily? (IF UNKNOWN, ENTER 99.)

|__|__|
AGE

★G11. Do you <u>currently</u> use smokeless tobacco at least once per day?

**YES**	**1**	**(G13)**
**NO**	**2**	

G12. How old were you when you last stopped using smokeless tobacco products daily? (IF UNKNOWN, ENTER 99.)

|__|__|
AGE

G13. In total, for how many years did you use smokeless tobacco products daily? Please do not count any years when you did <u>not</u> use these products. (IF UNKNOWN, ENTER 999.)

|__|__|__|
YEARS

G14. Thinking about all the years when you used smokeless tobacco products regularly, how many times do/did you usually use them in a single day? (IF UNKNOWN, ENTER 999.)

|__|__|__|
PER DAY

SECTION H. FAMILY HISTORY OF BREAST CANCER

Now I will ask you some questions about your female relatives.

★H1. How many full sisters do you have, both living and deceased? Please only include sisters with whom you share the same birth mother and father. Do not include adopted, stepsisters, or half sisters. (IF UNKNOWN, ENTER 99. IF NONE, ENTER 00.)

|__|__|
FULL SISTERS

★H2. How many half sisters do you have, both living and deceased? Please only include sisters with whom you share only one parent. Do not include adopted or stepsisters. (IF UNKNOWN, ENTER 99. IF NONE, ENTER 00.)

|__|__|
HALF SISTERS

★H3. How many daughters do you have? Be sure to include any who may have died, but do not include adopted, step, or foster daughters. (IF UNKNOWN, ENTER 99. IF NONE, ENTER 00.)

|___|___|
DAUGHTERS

★H4. Were any of your female relatives, specifically your birth mother, your full sisters, your half sisters, or your daughters, ever diagnosed with breast cancer? Please do not include adopted or step relatives.

YES.. 1
NO .... 2 (SECTION I)
DON'T KNOW....................................... 9 (SECTION I)

	FEMALE RELATIVE #1	FEMALE RELATIVE #2	FEMALE RELATIVE #3
**★H5. Which of your female relatives was diagnosed with breast cancer?**	**MOTHER.................. 1<br>FULL SISTER .... 2<br>MATERNAL HALF SISTER .... 3<br>PATERNAL HALF SISTER .... 4<br>DAUGHTER 5**	**MOTHER .... 1<br>FULL SISTER........... 2<br>MATERNAL HALF SISTER.................... 3<br>PATERNAL HALF SISTER.................... 4<br>DAUGHTER............... 5**	**MOTHER.................... 1<br>FULL SISTER .... 2<br>MATERNAL HALF SISTER .... 3<br>PATERNAL HALF SISTER .... 4<br>DAUGHTER 5**
H6. How old was she when this was first diagnosed? (IF UNKNOWN, ENTER 99.)	|__|__| AGE	|__|__| AGE	|__|__| AGE

SECTION I. ENVIRONMENTAL EXPOSURES

THE GRID SHOULD BE COMPLETED BY ASKING QUESTIONS ACROSS EACH ROW BEFORE MOVING ON TO THE NEXT ROW.

Now I will ask you some questions about anti-mosquito treatments and malarial controls.

★a. **Have you ever used :**	★b. **How often did/do you typically use this product?**	c. Where do you use this product? (SELECT ALL THAT APPLY.)	d. Do you use this product overnight?
★**I1. An insecticide-treated net (ITN)?**<br>**YES .... 1**<br>**NO .... 2 (I2)**<br>**DON'T KNOW . 9 (I2)**	**DAILY .... 1**<br>**WEEKLY .... 2**<br>**MONTHLY .... 3**<br>**<ONCE A MONTH................. 4**	INDOORS, BEDROOM OR SLEEPING AREA ...... 1<br>INDOORS, LIVING AREAS .. 2<br>OUTDOORS 3	YES .... 1<br>NO 2
★**I2. Repellent room spray?**<br>**YES .... 1**<br>**NO .... 2 (I3)**<br>**DON'T KNOW . 9 (I3)**	**DAILY .... 1**<br>**WEEKLY .... 2**<br>**MONTHLY .... 3**<br>**<ONCE A MONTH................. 4**	INDOORS, BEDROOM OR SLEEPING AREA ...... 1<br>INDOORS, LIVING AREAS .. 2	YES .... 1<br>NO 2
★**I3. Repellent skin cream?**<br>**YES .... 1**<br>**NO .... 2 (I4)**<br>**DON'T KNOW . 9 (I4)**	**DAILY .... 1**<br>**WEEKLY .... 2**<br>**MONTHLY .... 3**<br>**<ONCE A MONTH................. 4**	N/A	N/A
★**I4. A mosquito coil?**<br>**YES .... 1**<br>**NO .... 2 (I5)**<br>**DON'T KNOW . 9 (I5)**	**DAILY .... 1**<br>**WEEKLY .... 2**<br>**MONTHLY .... 3**<br>**<ONCE A MONTH................. 4**	INDOORS, BEDROOM OR SLEEPING AREA ...... 1<br>INDOORS, LIVING AREAS .. 2<br>OUTDOORS 3	N/A
★**I5. Other mosquito control products? (SPECIFY)**<br>________________<br>**YES .... 1**<br>**NO .... 2 (I6)**<br>**DON'T KNOW . 9 (I6)**	**DAILY .... 1**<br>**WEEKLY .... 2**<br>**MONTHLY .... 3**<br>**<ONCE A MONTH................. 4**	INDOORS, BEDROOM OR SLEEPING AREA ...... 1<br>INDOORS, LIVING AREAS .. 2<br>OUTDOORS 3	YES .... 1<br>NO 2

I6. Have you ever had an episode of malaria in which you had an extreme fever and intense sweating?

YES.. 1
NO 2 (I8)
DON'T KNOW 9 (I8)

★**I7. In the past <u>year</u>, how many episodes of malaria with extreme fever and intense sweating have you had?**

NONE 1
1 TO 2.. 2
3 TO 4.. 3
5 OR MORE 4
DON'T KNOW 9

Now I'm going to ask you about certain cosmetic and beauty products that you have used in the past.

★I8. Have you ever used a soap, cream, or other product to lighten or brighten your skin?

YES.. 1
NO 2 (I15)
DON'T KNOW 9 (I15)

I9. How old were you when you first used a product to lighten or brighten your skin? (IF UNKNOWN, ENTER 99.)

|__|__|
AGE

★I10. Do you currently use any product to lighten or brighten your skin?

YES.. 1 (I12)
NO 2

I11. How old were you when you last used any product to lighten or brighten your skin? (IF UNKNOWN, ENTER 99.)

|__|__|
AGE

I12. How long have you used/did you use any product to lighten or brighten your skin? Please do not include years when you did not use a product to lighten or brighten your skin. (IF UNKNOWN, ENTER 99.)

|__|__| MONTHS............................ 1
YEARS................................ 2

I13. When you use/used the skin lightener or brightener, how often do/did you usually apply it?

|__|__|__| PER: DAY........................ 1
TIMES WEEK..................... 2
MONTH 3

★I14. When you use/used the skin lightener or brightener, where do/did you apply it? (RECORD ALL THAT APPLY.)

FACE.. 1
ARMS 2
CHEST.. 3
ALL OVER BODY 4
OTHER (SPECIFY) 5

____

I15. Have you ever used a perming cream or relaxer to straighten your hair?

YES..	1	
NO	2	(SECTION J)
DON'T KNOW	9	(SECTION J)

I16. How old were you when you first used one of these products to straighten your hair? (IF UNKNOWN, ENTER 99.)

|__|__|
AGE

I17. Do you currently use a perming cream or relaxer to straighten your hair?

YES..	1	(I19)
NO	2	

I18. How old were you when you last used a perming cream or relaxer to straighten your hair? (IF UNKNOWN, ENTER 99.)

|__|__|
AGE

I19. How long have you used/did you use a perming cream or relaxer to straighten your hair? Please do not include years when you did not use them. (IF UNKNOWN, ENTER 99.)

|__|__|	MONTHS............................	1
	YEARS................................	2

I20. Which type of hair straightening products do/did you use most often? (<u>RECORD ONE ANSWER ONLY</u>.)

LYE...	1
NO-LYE	2
DON'T KNOW/DON'T REMEMBER	9

I21. How many times in your lifetime have you experienced burns (a break in the skin, not just tingling) during the application of these hair straighteners?

NEVER	1
1-2 TIMES...	2
3-4 TIMES...	3
5-9 TIMES...	4
10 OR MORE TIMES	5
DON'T KNOW/DON'T REMEMBER	9

SECTION J. OCCUPATION

Now I would like to ask you some questions about work that you may have done throughout your life.

★**J1. Have you ever lived on a farm, held a job, or taken on tasks or work on a farm, for a total of 6 months or longer? Please consider times when you were paid or unpaid and any work you may have done on your family's farm.**

YES.. 1
NO 2 (J16)

THE GRID SHOULD BE COMPLETED BY ASKING QUESTIONS DOWN EACH COLUMN BEFORE MOVING ON TO THE NEXT COLUMN.

Take a moment to think about all of the time you have spent living or working on a farm.

	**CIRCLE ONLY ONE CROP FOR EACH COLUMN.**			
	**a.**	**b.**	**c.**	**d.**
★**J2. Did you ever work or live on farmland that grew…..**<br>SHOW CARD J2	**Cashews .... 1**<br>**Cassava .... 2**<br>**Citrus .... 3**<br>**Cocoa.............. 4**<br>**Corn .... 5**<br>**Cotton .... 6**<br>**Mangoes .... 7**<br>**Onions............. 8**<br>**Plantains.......... 9**<br>**Rice .... 10**<br>**Sugarcane........ 11**<br>**Tomatoes......... 12**<br>**Yams .... 13**<br>**OTHER .... 14**<br>____________<br>**DON'T KNOW .. 15**	**Cashews.......... 1**<br>**Cassava .... 2**<br>**Citrus .... 3**<br>**Cocoa............... 4**<br>**Corn .... 5**<br>**Cotton .... 6**<br>**Mangoes .... 7**<br>**Onions .... 8**<br>**Plantains.......... 9**<br>**Rice..................10**<br>**Sugarcane ....11**<br>**Tomatoes.........12**<br>**Yams.................13**<br>**OTHER ....14**<br>____________<br>**DON'T KNOW...15**	**Cashews.......... 1**<br>**Cassava........... 2**<br>**Citrus............... 3**<br>**Cocoa .... 4**<br>**Corn................. 5**<br>**Cotton.............. 6**<br>**Mangoes.......... 7**<br>**Onions............. 8**<br>**Plantains .... 9**<br>**Rice.................. 10**<br>**Sugarcane........ 11**<br>**Tomatoes .... 12**<br>**Yams................ 13**<br>**OTHER............. 14**<br>____________<br>**DON'T KNOW... 15**	**Cashews .... 1**<br>**Cassava .... 2**<br>**Citrus............... 3**<br>**Cocoa .... 4**<br>**Corn.................. 5**<br>**Cotton .... 6**<br>**Mangoes .... 7**<br>**Onions............. 8**<br>**Plantains.......... 9**<br>**Rice .... 10**<br>**Sugarcane........ 11**<br>**Tomatoes......... 12**<br>**Yams .... 13**<br>**OTHER .... 14**<br>____________<br>**DON'T KNOW... 15**
J3. At what ages did you work or live on the farm that grew the [CROP]? (IF UNKNOWN, ENTER 99.)	|__|__| AGE<br>to<br>|__|__| AGE	|__|__| AGE<br>to<br>|__|__| AGE	|__|__| AGE<br>to<br>|__|__| AGE	|__|__| AGE<br>to<br>|__|__| AGE
★**J4. An herbicide or weedicide is a chemical applied to fields to kill weeds. Was an herbicide ever used on the [CROP]?**	**YES................1**<br>**NO..................2 (J8)**<br>**DON'T KNOW..9 (J8)**	**YES................ 1**<br>**NO.................. 2 (J8)**<br>**DON'T KNOW. 9 (J8)**	**YES................ 1**<br>**NO.................. 2 (J8)**<br>**DON'T KNOW. 9 (J8)**	**YES................ 1**<br>**NO.................. 2 (J8)**<br>**DON'T KNOW. 9 (J8)**
★**J5. Did you ever apply the herbicide or weedicide to the crop?**	**YES................1**<br>**NO..................2**<br>**DON'T KNOW..9**	**YES................ 1**<br>**NO.................. 2**<br>**DON'T KNOW. 9**	**YES................ 1**<br>**NO.................. 2**<br>**DON'T KNOW. 9**	**YES................ 1**<br>**NO.................. 2**<br>**DON'T KNOW. 9**
J6. How many years in total were herbicides or weedicides used on the [CROP] while you were working/living on the farm? (IF UNKNOWN, ENTER 99.)	|__|__| YEARS	|__|__| YEARS	|__|__| YEARS	|__|__| YEARS

J7. Do you know the brand name of the herbicide or weedicide most commonly used on the [CROP]? (IF UNKNOWN, ENTER 99.)	________ BRAND NAME	________ BRAND NAME	________ BRAND NAME	________ BRAND NAME
★**J8. An insecticide is a chemical applied to fields to kill insects, pests, or mites. Was an insecticide ever used on the [CROP]?**	**YES................1<br>NO..................2 (J12)<br>DON'T KNOW..9 (J12)**	**YES................ 1<br>NO.................. 2 (J12)<br>DON'T KNOW. 9 (J12)**	**YES................ 1<br>NO.................. 2 (J12)<br>DON'T KNOW. 9 (J12)**	**YES................ 1<br>NO.................. 2 (J12)<br>DON'T KNOW. 9 (J12)**
★**J9. Did you ever apply the insecticide to the crop?**	**YES................1<br>NO..................2<br>DON'T KNOW..9**	**YES................ 1<br>NO.................. 2<br>DON'T KNOW. 9**	**YES................ 1<br>NO.................. 2<br>DON'T KNOW. 9**	**YES................ 1<br>NO.................. 2<br>DON'T KNOW. 9**
J10. How many years in total were insecticides used on the [CROP] while you were working/living on the farm? (IF UNKNOWN, ENTER 99.)	|__|__| YEARS	|__|__| YEARS	|__|__| YEARS	|__|__| YEARS
J11. Do you know the brand name of the insecticide most commonly used on the [CROP]? (IF UNKNOWN, ENTER 99.)	________ BRAND NAME	________ BRAND NAME	________ BRAND NAME	________ BRAND NAME
★**J12. A fungicide is a chemical applied to fields to prevent or kill mold and spores. Was a fungicide ever used on the [CROP]?**	**YES................1<br>NO..................2 (J16)<br>DON'T KNOW..9 (J16)**	**YES................ 1<br>NO.................. 2 (J16)<br>DON'T KNOW. 9 (J16)**	**YES................ 1<br>NO.................. 2 (J16)<br>DON'T KNOW. 9 (J16)**	**YES................ 1<br>NO.................. 2 (J16)<br>DON'T KNOW. 9 (J16)**
★**J13. Did you ever apply the fungicide to the crop?**	**YES................1<br>NO..................2<br>DON'T KNOW..9**	**YES................ 1<br>NO.................. 2<br>DON'T KNOW. 9**	**YES................ 1<br>NO.................. 2<br>DON'T KNOW. 9**	**YES................ 1<br>NO.................. 2<br>DON'T KNOW. 9**
J14. How many years in total were fungicides used on the [CROP] while you were working/living on the farm? (IF UNKNOWN, ENTER 99.)	|__|__| YEARS	|__|__| YEARS	|__|__| YEARS	|__|__| YEARS
J15. Do you know the brand name of the fungicide most commonly used on the [CROP]? (IF UNKNOWN, ENTER 99.)	________ BRAND NAME	________ BRAND NAME	________ BRAND NAME	________ BRAND NAME

IF MORE THAN 4 CROPS, USE ADDITIONAL PAGES TO RECORD DETAILS ABOUT EACH ADDITIONAL CROP (FOUND AT END OF THIS QUESTIONNAIRE).

J16. Have you ever worked as a cosmetologist for a total of 6 months or longer?

YES.. 1
NO 2 (J18)

J17. At what ages did you work as a cosmetologist? (IF UNKNOWN, ENTER 99.)

|__|__| to |__|__|
AGE AGE

J18. Have you ever worked as a hairdresser for a total of 6 months or longer?

YES.. 1
NO 2 (J20)

J19. At what ages did you work as a hairdresser? (IF UNKNOWN, ENTER 99.)

|__|__| to |__|__|
AGE AGE

J20. Have you ever worked as a petrol or gas station attendant, for a total of 6 months or longer?

YES.. 1
NO .... 2 (SECTION K)

J21. At what ages did you work as a petrol or gas station attendant? (IF UNKNOWN, ENTER 99.)

|__|__| to |__|__|
AGE AGE

SECTION K. HEALTH CARE UTILIZATION AND MEDICAL HISTORY

Now I will ask you some questions concerning your medical care and other things you do to maintain your health. By medical care, I mean visits to a medical doctor or nurse at a hospital, clinic, other medical facility, or traditional healer for your health concerns. Traditional healers include fetish priests, herbalists, and faith healers.

★K1. Who do you usually seek help from when you are ill? Please do not include visits made related to the health of friends or family members.

DOCTOR 1
NURSE 2
TRADITIONAL HEALER
(FETISH PRIEST, HERBALIST, FAITH HEALER) 3 (K7)
DO NOT SEEK HELP 4 (K12)
OTHER (SPECIFY)... 5 (K6)

____

K2. Where do you see this doctor or nurse? Is it a private or government operated hospital or clinic? (RECORD ONE ANSWER ONLY.)

PRIVATE 1
GOVERNMENT..................................... 2
DON'T KNOW 9

K3. On average, how often do you visit a doctor or nurse? (IF UNKNOWN, ENTER 999.)

|__|__|__| PER: MONTH 1
TIMES YEAR 2
LIFETIME............... 3

K4. When you see a doctor or nurse, do you usually have your weight measured?

YES.. 1
NO 2
DON'T KNOW 9

K5. When you see a doctor or nurse, do you usually have your blood pressure measured?

YES.. 1
NO 2
DON'T KNOW 9

★K6. In addition to the places you usually go for health care, have you ever seen a traditional healer about your health? This is someone who is not a doctor or nurse. Traditional healers include fetish priests, herbalists, and faith healers. Please do not count visits you made related to the health of other friends or family members.

YES.. 1
NO 2 (K9)
DON'T KNOW....................................... 9 (K9)

K7. What type of traditional healer was he/she?

FETISH PRIEST	1
HERBALIST	2
FAITH HEALER	3
OTHER (SPECIFY) ______________	4
DON'T KNOW	9

K8. On average, how often do you visit a traditional healer about your health? Please do not count visits related to the health of others. (IF UNKNOWN, ENTER 999.)

|__|__|__| TIME(S)	PER:	MONTH	1
		YEAR	2
		LIFETIME	3

K9. Have you ever taken any traditional medications or treatments?

YES	1	
NO	2	(K11)
DON'T KNOW	9	(K11)

K10. On average, how often did/do you usually take traditional medications or treatments? (IF UNKNOWN, ENTER 999.)

|__|__|__| TIMES	PER:	DAY	1
		MONTH	2
		YEAR	3
		LIFETIME	4

★K11. How long does it usually take you to travel from your home to the place where you are most likely to seek health care? (IF UNKNOWN, ENTER 999.)

**|__|__|__| NUMBER**	**MINUTES**	**1**
	**HOURS**	**2**
	**DAYS**	**3**

K12. What difficulties or hardships do you face when seeking health care? (CHOOSE ALL THAT APPLY.)

SHOW CARD K12

COST (MEDICAL BILLS, MEDICINES)	1
DISTANCE/TRANSPORTATION	2
TIME AWAY FROM FAMILY	3
TIME AWAY FROM WORK	4
FINDING A PLACE TO STAY WHEN RECEIVING CARE	5
TOO SICK TO TRAVEL	6
FEAR OF DOCTORS AND/OR HOSPITALS	7
OTHER REASON (SPECIFY) ______________	8
NO DIFFICULTIES	9

K13. Have you ever been told that you have diabetes (high sugar in blood or urine)?

YES..	1	
NO	2	(K17)
DON'T KNOW	9	(K17)

K14. How old were you when you were <u>first</u> told you had diabetes? (IF UNKNOWN, ENTER 99.)

|__|__|
AGE

K15. Was this during pregnancy only?

YES..	1
NO	2
DON'T KNOW	9

K16. What treatment have you had for your diabetes? (RECORD ALL THAT APPLY.)

INSULIN...	1
PILLS	2
DIET/WEIGHT LOSS	3
OTHER (SPECIFY)	4
________________________________	
NO TREATMENT	5
DON'T KNOW	9

K17. A mammogram is an x-ray of the breasts taken by a machine. Prior to one year ago, did you ever have a mammogram?

YES..	1	
NO	2	(K19)
DON'T KNOW	9	(K19)

K18. How old were you when you had your first mammogram? (IF UNKNOWN, ENTER 99.)

|__|__|
AGE

K19. An ultrasound examination uses sound waves instead of radiation to examine the body. Prior to one year ago, did you ever have an ultrasound of your breast?

YES..	1	
NO	2	(K21)
DON'T KNOW	9	(K21)

K20. How old were you when you first had an ultrasound of your breast? (IF DON'T KNOW, ENTER 99.)

|__|__|
AGE

K21. A breast exam is when your breasts and underarms are checked for lumps, rashes or dimpling, and/or differences in size or shape. Prior to one year ago, did you ever have your breasts examined by a health professional or traditional healer?

YES..	1	
NO	2	(K23)
DON'T KNOW	9	(K23)

K22. How old were you when you had your first breast exam? (IF DON'T KNOW, ENTER 99.)

|__|__|
AGE

★K23. A breast biopsy is the removal of breast tissue or cells for the purpose of making a diagnosis. Prior to one year ago, did you ever have a breast biopsy?

**YES..**	**1**	
**NO ....**	**2**	**(SECTION L)**
**DON'T KNOW ....**	**9**	**(SECTION L)**

K24. How old were you when you had your <u>first</u> breast biopsy? (IF DON'T KNOW, ENTER 99.)

|__|__|
AGE

★K25. Was breast cancer diagnosed as a result of your <u>first</u> biopsy?

**YES..**	**1**
**NO ....**	**2**
**DON'T KNOW ....**	**9**

SECTION L. PHYSICAL ACTIVITY

THE GRID SHOULD BE COMPLETED BY ASKING QUESTIONS ACROSS EACH ROW BEFORE MOVING ON TO THE NEXT ROW.

Next, I will ask you questions about <u>your usual level</u> of physical activity. I will begin by asking you how often you usually performed different activities when you were 12 or 13 years old. Then I will ask you about how much time you <u>usually</u> spent doing these same activities 5 years ago. To help remember the different time periods in your life, think about where you were living, what you were doing (in school or working), and what your family was doing.

When you were 12 or 13 years old, did you:		b. How much time <u>per week</u> did you spend doing this?
L1. Perform light household tasks (for example, washing dishes, sweeping, mopping)?<br>SHOW CARD L1 & L6	a. YES .... 1<br>NO 2 (L2)	5 MINUTES .... 01<br>15 MINUTES .... 02<br>30 MIN .... 03<br>1 HOUR .... 04<br>1 HR 30 MIN .... 05<br>2-3 HOURS .... 06<br>4-6 HOURS .... 07<br>7-10 HOURS .... 08<br>>10 HRS .... 09<br>DON'T KNOW 99
L2. Perform heavy household tasks (for example, pounding cassava, vigorous scrubbing or cleaning)?<br>SHOW CARD L2 & L7	a. YES .... 1<br>NO 2 (L3)	5 MINUTES .... 01<br>15 MINUTES .... 02<br>30 MIN .... 03<br>1 HOUR .... 04<br>1 HR 30 MIN .... 05<br>2-3 HOURS .... 06<br>4-6 HOURS .... 07<br>7-10 HOURS .... 08<br>>10 HRS .... 09<br>DON'T KNOW 99
L3. Lift or carry heavy loads (for example, fetching water)?<br>SHOW CARD L3 & L8	a. YES .... 1<br>NO 2 (L4)	5 MINUTES .... 01<br>15 MINUTES .... 02<br>30 MIN .... 03<br>1 HOUR .... 04<br>1 HR 30 MIN .... 05<br>2-3 HOURS .... 06<br>4-6 HOURS .... 07<br>7-10 HOURS .... 08<br>>10 HRS .... 09<br>DON'T KNOW 99
L4. Run, play football, or play other games that involved continuous movement?<br>SHOW CARD L4 & L11	a. YES .... 1<br>NO 2 (L5)	5 MINUTES .... 01<br>15 MINUTES .... 02<br>30 MIN .... 03<br>1 HOUR .... 04<br>1 HR 30 MIN .... 05<br>2-3 HOURS .... 06<br>4-6 HOURS .... 07<br>7-10 HOURS .... 08<br>>10 HRS .... 09<br>DON'T KNOW 99

When you were 12 or 13 years old, did you:		b. How much time per week did you spend doing this?
L5. Walk, including going to and from school or work, to market, or to a neighbor or relative's house?	a. YES .... 1<br>NO 2 (L6)	5 MINUTES .... 01<br>15 MINUTES .... 02<br>30 MIN .... 03<br>1 HOUR .... 04<br>1 HR 30 MIN .... 05<br>2-3 HOURS .... 06<br>4-6 HOURS .... 07<br>7-10 HOURS .... 08<br>>10 HRS .... 09<br>DON'T KNOW 99

Five years ago when you were___ years old, did you:		b. How much time per week did you spend doing this?
L6. Perform light household tasks (for example, washing dishes, sweeping, mopping)?<br>SHOW CARD L1 & L6	a. YES .... 1<br>NO 2 (L7)	5 MINUTES .... 01<br>15 MINUTES .... 02<br>30 MIN .... 03<br>1 HOUR .... 04<br>1 HR 30 MIN .... 05<br>2-3 HOURS .... 06<br>4-6 HOURS .... 07<br>7-10 HOURS .... 08<br>>10 HRS .... 09<br>DON'T KNOW 99
L7. Perform heavy household tasks (for example, pounding cassava, vigorous scrubbing or cleaning)?<br>SHOW CARD L2 & L7	a. YES .... 1<br>NO 2 (L8)	5 MINUTES .... 01<br>15 MINUTES .... 02<br>30 MIN .... 03<br>1 HOUR .... 04<br>1 HR 30 MIN .... 05<br>2-3 HOURS .... 06<br>4-6 HOURS .... 07<br>7-10 HOURS .... 08<br>>10 HRS .... 09<br>DON'T KNOW 99
L8. Lift or carry heavy loads (for example, fetching water)?<br>SHOW CARD L3 & L8	a. YES .... 1<br>NO 2 (L9)	5 MINUTES .... 01<br>15 MINUTES .... 02<br>30 MIN .... 03<br>1 HOUR .... 04<br>1 HR 30 MIN .... 05<br>2-3 HOURS .... 06<br>4-6 HOURS .... 07<br>7-10 HOURS .... 08<br>>10 HRS .... 09<br>DON'T KNOW 99

Five years ago when you were___ years old, did you:		b. How much time per week did you spend doing this?
L9. Bathe and/or care for children?<br><br>SHOW CARD L9	a. YES..................1<br>NO 2 (L10)	5 MINUTES...................... 01<br>15 MINUTES.................... 02<br>30 MIN.............................. 03<br>1 HOUR............................ 04<br>1 HR 30 MIN .... 05<br>2-3 HOURS...................... 06<br>4-6 HOURS...................... 07<br>7-10 HOURS.................... 08<br>>10 HRS .... 09<br>DON'T KNOW.................. 99
L10. Bathe and/or care for elders?<br><br>SHOW CARD L10	a. YES..................1<br>NO 2 (L11)	5 MINUTES...................... 01<br>15 MINUTES.................... 02<br>30 MIN.............................. 03<br>1 HOUR............................ 04<br>1 HR 30 MIN .... 05<br>2-3 HOURS...................... 06<br>4-6 HOURS...................... 07<br>7-10 HOURS.................... 08<br>>10 HRS .... 09<br>DON'T KNOW.................. 99
L11. Run, play football, or play other games that involved continuous movement?<br><br>SHOW CARD L4 & L11	a. YES..................1<br>NO 2 (L12)	5 MINUTES...................... 01<br>15 MINUTES.................... 02<br>30 MIN.............................. 03<br>1 HOUR............................ 04<br>1 HR 30 MIN .... 05<br>2-3 HOURS...................... 06<br>4-6 HOURS...................... 07<br>7-10 HOURS.................... 08<br>>10 HRS .... 09<br>DON'T KNOW.................. 99
L12. Walk, including going to and from school or work, to market, or to a neighbor or relative's house?	a. YES..................1<br>NO 2	5 MINUTES...................... 01<br>15 MINUTES.................... 02<br>30 MIN.............................. 03<br>1 HOUR............................ 04<br>1 HR 30 MIN .... 05<br>2-3 HOURS...................... 06<br>4-6 HOURS...................... 07<br>7-10 HOURS.................... 08<br>>10 HRS .... 09<br>DON'T KNOW.................. 99

IF RESPONDENT IS A CASE IN THIS STUDY, THEN CONTINUE TO SECTION M.
IF RESPONDENT IS A CONTROL IN THIS STUDY, THEN GO TO QUESTIONNAIRE END.

SECTION M. SUPPLEMENTARY QUESTIONS FOR WOMEN ENROLLED AS A CASE IN THE STUDY

Now I am going to ask you some questions related to changes you may have experienced before your breast biopsy or around the time of your breast biopsy.

★M1. When did you first notice a problem with your breast(s)? (RECORD MONTH IN 3-LETTER ABBREVIATIONS LIKE JAN, FEB, MAR,…DEC. IF UNKNOWN, ENTER XXX FOR MONTH, 9999 FOR YEAR.)

|__|__|__| / |__|__|__|__|
MONTH YEAR

AGE ESTIMATE _______

★M2. What problem(s) did you notice first? (SELECT ALL THAT APPLY.)

LUMP OR MASS	1
PAIN OR TENDERNESS	2
NIPPLE DISCHARGE	3
SKIN ULCERATION OR RASH	4
INFECTION	5
SKIN DIMPLING	6
CHANGE IN SIZE OF BREAST	7
OTHER (SPECIFY) ________________	8
DON'T KNOW	9

★M3. Which breast was affected by this/these problem(s)?

LEFT ONLY	1
RIGHT ONLY	2
BOTH	3
DON'T KNOW	4

★M4. When did you first seek help or treatment related to this/these problem(s)? (RECORD MONTH IN 3-LETTER ABBREVIATIONS LIKE JAN, FEB, MAR,…DEC. IF UNKNOWN, ENTER XXX FOR MONTH, 9999 FOR YEAR.)

|__|__|__| / |__|__|__|__|
MONTH YEAR

AGE ESTIMATE _______

★M5. Who did you first see for help or treatment related to this/these problem(s)?

DOCTOR	1
NURSE	2
TRADITIONAL HEALER (FETISH PRIEST, HERBALIST, FAITH HEALER)	3
DID NOT SEEK HELP	4
OTHER (SPECIFY) ________________	5

<u>QUESTIONNAIRE END</u>: This is the end of the interview. Thank you very much for your time and patience. Your participation is very important to this study.

SECTION N. QUALITY OF INTERVIEW

★N1. **End time of interview:** |__|__| : |__|__| Use 24 hr clock. Example: 14:00

★N2. **Location of the interview was:**

Hospital	1
Clinic	2
Home	3
Other (SPECIFY)	4

____

★N3. **Interview outcome code:**

Completed all	1
Critical questions only	2
Partially completed	3
Partially refused	4

★N4. **Participant's cooperation was:**

Very Good	1
Good	2
Fair	3
Poor	4

★N5. **The overall quality of this interview was:**

High quality	1	(N7)
Generally reliable	2	(N7)
Questionable	3	(N7)
Unsatisfactory	4	

★N6. **If the interview was unsatisfactory, what were the three main reasons?**

|__|__| |__|__| |__|__|

CODES FOR MAIN REASON:

Did not know enough information regarding the topic	01
Did not want to be more specific	02
Did not understand the questions	03
Was bored or uninterested	04
Was upset, depressed or angry	05
Had poor hearing or speech	06
Was confused or distracted by frequent interruptions	07
Was inhibited by others around her	08
Was embarrassed by the subject matter	09
Was emotionally unstable	10
Was physically ill	11
Was too tired to complete	12
Other (SPECIFY)	13

____

★N7. **Were there any distractions during the interview?**

YES	1	
NO	2	(N9)

★N8. **Describe the distraction:**

____

____

★N9. **Other comments from interviewer:**

____

____

____

★N10. **In what language was the interview spoken?**

English	1
Twi	2
Combination of English and Twi	3
Other (SPECIFY)	4

____

PLEASE REVIEW THIS QUESTIONNAIRE FOR COMPLETENESS
AND ACCURACY AFTER ADMINISTRATION.
THANK YOU.

IF MORE THAN 6 PREGNANCIES, USE THESE ADDITIONAL PAGES TO RECORD DETAILS ABOUT EACH ADDITIONAL PREGNANCY. THE GRID SHOULD BE COMPLETED BY ASKING QUESTIONS DOWN EACH COLUMN BEFORE MOVING ON TO THE NEXT COLUMN.

B2. Did this pregnancy end as a live birth, stillbirth, miscarriage, abortion, tubal pregnancy, or ectopic pregnancy?

g. PREGNANCY	h. PREGNANCY	i. PREGNANCY
LIVE BIRTH...................... 2<br>MULTIPLE BIRTHS .... 3<br>STILLBIRTH<br>(≥5 MONTHS) .... 4<br>MISCARRIAGE<br>(<5 MONTHS) .... 5<br>TUBAL OR ECTOPIC<br>PREGNANCY .... 6<br>INDUCED ABORTION ..... 7<br>OTHER (SPECIFY).......... 8<br>______________________	LIVE BIRTH..................... 2<br>MULTIPLE BIRTHS......... 3<br>STILLBIRTH<br>(≥5 MONTHS).............. 4<br>MISCARRIAGE<br>(<5 MONTHS).............. 5<br>TUBAL OR ECTOPIC<br>PREGNANCY............... 6<br>INDUCED ABORTION .... 7<br>OTHER (SPECIFY) .... 8<br>______________________	LIVE BIRTH .... 2<br>MULTIPLE BIRTHS .... 3<br>STILLBIRTH<br>(≥5 MONTHS) .... 4<br>MISCARRIAGE<br>(<5 MONTHS) .... 5<br>TUBAL OR ECTOPIC<br>PREGNANCY .... 6<br>INDUCED ABORTION..... 7<br>OTHER (SPECIFY).......... 8<br>______________________

B3. Please tell me when your pregnancy ended or your baby was born. (RECORD RESPONDENT'S AGE ESTIMATE IF SHE CANNOT REMEMBER MONTH AND YEAR.)

|__|__|__| / |__|__|__|__|__| MONTH YEAR<br>MOTHER'S AGE<br>ESTIMATE _______	|__|__|__| / |__|__|__|__|__| MONTH YEAR<br>MOTHER'S AGE<br>ESTIMATE _______	|__|__|__| / |__|__|__|__|__| MONTH YEAR<br>MOTHER'S AGE<br>ESTIMATE _______

B4. ONLY IF MULTIPLE BIRTHS: How many live and still birth babies resulted from this pregnancy?

LIVE BIRTHS: |__|__| STILL BIRTHS: |__|__|	LIVE BIRTHS: |__|__| STILL BIRTHS: |__|__|	LIVE BIRTHS: |__|__| STILL BIRTHS: |__|__|

B5. ONLY IF LIVE BIRTH: Did you breastfeed this baby/these babies?

YES................................. 1<br>NO................................... 2<br>(IF NO, GO TO NEXT PREGNANCY)	YES .... 1<br>NO .... 2<br>(IF NO, GO TO NEXT PREGNANCY)	YES................................. 1<br>NO .... 2<br>(IF NO, GO TO NEXT PREGNANCY)

B6. ONLY IF BREAST FED: How long did you breastfeed this baby/these babies? (IF LESS THAN ONE MONTH, CODE AS "0" MONTHS.)

|__|__|__| MONTHS	|__|__|__| MONTHS	|__|__|__| MONTHS

B2. Did this pregnancy end as a live birth, stillbirth, miscarriage, abortion, tubal pregnancy, or ectopic pregnancy?

j. PREGNANCY	k. PREGNANCY	l. PREGNANCY
LIVE BIRTH...................... 2<br>MULTIPLE BIRTHS .... 3<br>STILLBIRTH<br>(≥5 MONTHS) .... 4<br>MISCARRIAGE<br>(<5 MONTHS) .... 5<br>TUBAL OR ECTOPIC<br>PREGNANCY .... 6<br>INDUCED ABORTION ..... 7<br>OTHER (SPECIFY).......... 8<br>______________________	LIVE BIRTH .... 2<br>MULTIPLE BIRTHS......... 3<br>STILLBIRTH<br>(≥5 MONTHS).............. 4<br>MISCARRIAGE<br>(<5 MONTHS).............. 5<br>TUBAL OR ECTOPIC<br>PREGNANCY............... 6<br>INDUCED ABORTION .... 7<br>OTHER (SPECIFY) .... 8<br>______________________	LIVE BIRTH .... 2<br>MULTIPLE BIRTHS .... 3<br>STILLBIRTH<br>(≥5 MONTHS) .... 4<br>MISCARRIAGE<br>(<5 MONTHS) .... 5<br>TUBAL OR ECTOPIC<br>PREGNANCY .... 6<br>INDUCED ABORTION..... 7<br>OTHER (SPECIFY).......... 8<br>______________________

B3. Please tell me when your pregnancy ended or your baby was born. (RECORD RESPONDENT'S AGE ESTIMATE IF SHE CANNOT REMEMBER MONTH AND YEAR.)

|__|__|__| / |__|__|__|__| MONTH YEAR<br>MOTHER'S AGE<br>ESTIMATE _______	|__|__|__| / |__|__|__|__| MONTH YEAR<br>MOTHER'S AGE<br>ESTIMATE _______	|__|__|__| / |__|__|__|__| MONTH YEAR<br>MOTHER'S AGE<br>ESTIMATE _______

B4. ONLY IF MULTIPLE BIRTHS: How many live and still birth babies resulted from this pregnancy?

LIVE BIRTHS: |__|__| STILL BIRTHS: |__|__|	LIVE BIRTHS: |__|__| STILL BIRTHS: |__|__|	LIVE BIRTHS: |__|__| STILL BIRTHS: |__|__|

B5. ONLY IF LIVE BIRTH: Did you breastfeed this baby/these babies?

YES................................. 1<br>NO................................... 2<br>(IF NO, GO TO NEXT PREGNANCY)	YES .... 1<br>NO .... 2<br>(IF NO, GO TO NEXT PREGNANCY)	YES................................. 1<br>NO .... 2<br>(IF NO, GO TO NEXT PREGNANCY)

B6. ONLY IF BREAST FED: How long did you breastfeed this baby/these babies? (IF LESS THAN ONE MONTH, CODE AS "0" MONTHS.)

|__|__|__| MONTHS	|__|__|__| MONTHS	|__|__|__| MONTHS

IF MORE THAN 4 CROPS, USE THESE ADDITIONAL PAGES TO RECORD DETAILS ABOUT EACH ADDITIONAL CROP.

	CIRCLE ONLY ONE CROP FOR EACH COLUMN.			
	**e.**	**f.**	**g.**	**h.**
★**J2. Did you ever work or live on farmland that grew…..**<br><br>SHOW CARD J2	**Cashews .... 1**<br>**Cassava .... 2**<br>**Citrus .... 3**<br>**Cocoa.............. 4**<br>**Corn .... 5**<br>**Cotton .... 6**<br>**Mangoes .... 7**<br>**Onions............. 8**<br>**Plantains.......... 9**<br>**Rice .... 10**<br>**Sugarcane........ 11**<br>**Tomatoes......... 12**<br>**Yams .... 13**<br>**OTHER .... 14**<br>________________<br>**DON'T KNOW .. 15**	**Cashews.......... 1**<br>**Cassava........... 2**<br>**Citrus .... 3**<br>**Cocoa............... 4**<br>**Corn .... 5**<br>**Cotton .... 6**<br>**Mangoes .... 7**<br>**Onions .... 8**<br>**Plantains........... 9**<br>**Rice..................10**<br>**Sugarcane ....11**<br>**Tomatoes.........12**<br>**Yams.................13**<br>**OTHER..............14**<br>________________<br>**DON'T KNOW...15**	**Cashews.......... 1**<br>**Cassava........... 2**<br>**Citrus............... 3**<br>**Cocoa .... 4**<br>**Corn................. 5**<br>**Cotton.............. 6**<br>**Mangoes.......... 7**<br>**Onions............. 8**<br>**Plantains .... 9**<br>**Rice.................. 10**<br>**Sugarcane........ 11**<br>**Tomatoes .... 12**<br>**Yams................ 13**<br>**OTHER............. 14**<br>________________<br>**DON'T KNOW... 15**	**Cashews .... 1**<br>**Cassava .... 2**<br>**Citrus............... 3**<br>**Cocoa.............. 4**<br>**Corn.................. 5**<br>**Cotton .... 6**<br>**Mangoes .... 7**<br>**Onions............. 8**<br>**Plantains.......... 9**<br>**Rice .... 10**<br>**Sugarcane........ 11**<br>**Tomatoes......... 12**<br>**Yams .... 13**<br>**OTHER .... 14**<br>________________<br>**DON'T KNOW... 15**
J3. At what ages did you work or live on the farm that grew the [CROP]? (IF UNKNOWN, ENTER 99.)	|__|__| AGE<br>to<br>|__|__| AGE	|__|__| AGE<br>to<br>|__|__| AGE	|__|__| AGE<br>to<br>|__|__| AGE	|__|__| AGE<br>to<br>|__|__| AGE
★**J4. An herbicide or weedicide is a chemical applied to fields to kill weeds. Was an herbicide ever used on the [CROP]?**	**YES................1**<br>**NO..................2 (J8)**<br>**DON'T KNOW..9 (J8)**	**YES............... 1**<br>**NO................. 2 (J8)**<br>**DON'T KNOW. 9 (J8)**	**YES............... 1**<br>**NO................. 2 (J8)**<br>**DON'T KNOW. 9 (J8)**	**YES............... 1**<br>**NO................. 2 (J8)**<br>**DON'T KNOW. 9 (J8)**
★**J5. Did you ever apply the herbicide or weedicide to the crop?**	**YES................1**<br>**NO..................2**<br>**DON'T KNOW..9**	**YES............... 1**<br>**NO................. 2**<br>**DON'T KNOW. 9**	**YES............... 1**<br>**NO................. 2**<br>**DON'T KNOW. 9**	**YES............... 1**<br>**NO................. 2**<br>**DON'T KNOW. 9**
J6. How many years in total were herbicides or weedicides used on the [CROP] while you were working/living on the farm? (IF UNKNOWN, ENTER 99.)	|__|__| YEARS	|__|__| YEARS	|__|__| YEARS	|__|__| YEARS
J7. Do you know the brand name of the herbicide or weedicide most commonly used on the [CROP]? (IF UNKNOWN, ENTER 99.)	________________<br>BRAND NAME	________________<br>BRAND NAME	________________<br>BRAND NAME	________________<br>BRAND NAME
★**J8. An insecticide is a chemical applied to fields to kill insects, pests, or mites. Was an insecticide ever used on the [CROP]?**	**YES................1**<br>**NO..................2 (J12)**<br>**DON'T KNOW..9 (J12)**	**YES............... 1**<br>**NO................. 2 (J12)**<br>**DON'T KNOW. 9 (J12)**	**YES............... 1**<br>**NO................. 2 (J12)**<br>**DON'T KNOW. 9 (J12)**	**YES............... 1**<br>**NO................. 2 (J12)**<br>**DON'T KNOW. 9 (J12)**

★**J9.**	**Did you ever apply the insecticide to the crop?**	**YES................1**<br>**NO..................2**<br>**DON'T KNOW..9**	**YES............... 1**<br>**NO................. 2**<br>**DON'T KNOW. 9**	**YES............... 1**<br>**NO................. 2**<br>**DON'T KNOW. 9**	**YES............... 1**<br>**NO................. 2**<br>**DON'T KNOW. 9**
J10.	How many years in total were insecticides used on the [CROP] while you were working/living on the farm? (IF UNKNOWN, ENTER 99.)	|__|__| YEARS	|__|__| YEARS	|__|__| YEARS	|__|__| YEARS
J11.	Do you know the brand name of the insecticide most commonly used on the [CROP]? (IF UNKNOWN, ENTER 99.)	______________<br>BRAND NAME	______________<br>BRAND NAME	______________<br>BRAND NAME	______________<br>BRAND NAME
★**J12.**	**A fungicide is a chemical applied to fields to prevent or kill mold and spores. Was a fungicide ever used on the [CROP]?**	**YES................1**<br>**NO..................2 (J16)**<br>**DON'T KNOW..9 (J16)**	**YES............... 1**<br>**NO................. 2 (J16)**<br>**DON'T KNOW. 9 (J16)**	**YES............... 1**<br>**NO................. 2 (J16)**<br>**DON'T KNOW. 9 (J16)**	**YES............... 1**<br>**NO................. 2 (J16)**<br>**DON'T KNOW. 9 (J16)**
★**J13.**	**Did you ever apply the fungicide to the crop?**	**YES................1**<br>**NO..................2**<br>**DON'T KNOW..9**	**YES............... 1**<br>**NO................. 2**<br>**DON'T KNOW. 9**	**YES............... 1**<br>**NO................. 2**<br>**DON'T KNOW. 9**	**YES............... 1**<br>**NO................. 2**<br>**DON'T KNOW. 9**
J14.	How many years in total were fungicides used on the [CROP] while you were working/living on the farm? (IF UNKNOWN, ENTER 99.)	|__|__| YEARS	|__|__| YEARS	|__|__| YEARS	|__|__| YEARS
J15.	Do you know the brand name of the fungicide most commonly used on the [CROP]? (IF UNKNOWN, ENTER 99.)	______________<br>BRAND NAME	______________<br>BRAND NAME	______________<br>BRAND NAME	______________<br>BRAND NAME

THIS PAGE INTENTIONALLY BLANK
